# Boron Neutron Capture Therapy at a Crossroads: Translational Gap and Emerging Delivery Agents

**DOI:** 10.1002/chem.202503533

**Published:** 2026-02-15

**Authors:** Christoph Selg, Evamarie Hey‐Hawkins

**Affiliations:** ^1^ Faculty of Chemistry Centre For Biotechnology and Biomedicine (BBZ) Institute of Bioanalytical Chemistry Leipzig University Leipzig Germany; ^2^ Department of Chemistry Babeș‐Bolyai University Cluj‐Napoca Romania

**Keywords:** drug delivery, nanoparticle, targeted boron agents, theranostics, translational radiotherapy

## Abstract

Boron neutron capture therapy (BNCT) combines molecular targeting with localized high linear energy transfer radiation, offering a highly selective treatment for refractory malignancies. Despite decades of research, all clinical BNCT trials to date have relied exclusively on the legacy agents boronophenylalanine (BPA) and sodium borocaptate (BSH), reflecting historical precedent and regulatory familiarity rather than optimal pharmacological performance. Recent advances in boron chemistry, nanotechnology, and molecular imaging have generated a new generation of boron delivery agents with improved tumor selectivity, multifunctionality, and theranostic potential, yet none have reached clinical translation. This review covers boron carriers reported since 2018, categorized as small molecules, boronated polymers and liposomes, boron‐conjugated biomolecules, and imaging‐guided or theranostic systems. Trends derived from bibliometric analysis reveal a shift toward nanoparticle‐based carriers, which offer modular architectures for simultaneous drug loading, targeting, and imaging. Among these, multifunctional nanoplatforms appear particularly promising for personalized BNCT, where in vivo imaging via PET, MRI, SPECT, or fluorescence can enable patient‐specific treatment planning and real‐time dosimetry. However, the translation of these innovative agents remains constrained by economic, regulatory, and logistical barriers. The field's next frontier will require harmonized dosimetry protocols, validated imaging standards, and coordinated industrial and academic investment to bridge the gap between chemistry and clinic. BNCT thus stands at a translational gap: sustained interdisciplinary collaboration could finally expand its clinical repertoire beyond BPA and BSH toward a new generation of targeted and image‐guided boron therapeutics.

## Graphical abstract

This review surveys recent advances in boron delivery agents for BNCT, emphasizing the shift from classical small molecules to multifunctional nanocarriers and theranostic systems. By integrating targeting, imaging, and therapy, next‐generation boron compounds aim to bridge the gap between (bio)chemical innovation and clinical translation.

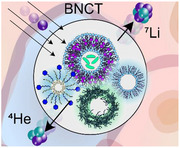

## 1 Introduction

**FIGURE 1:**
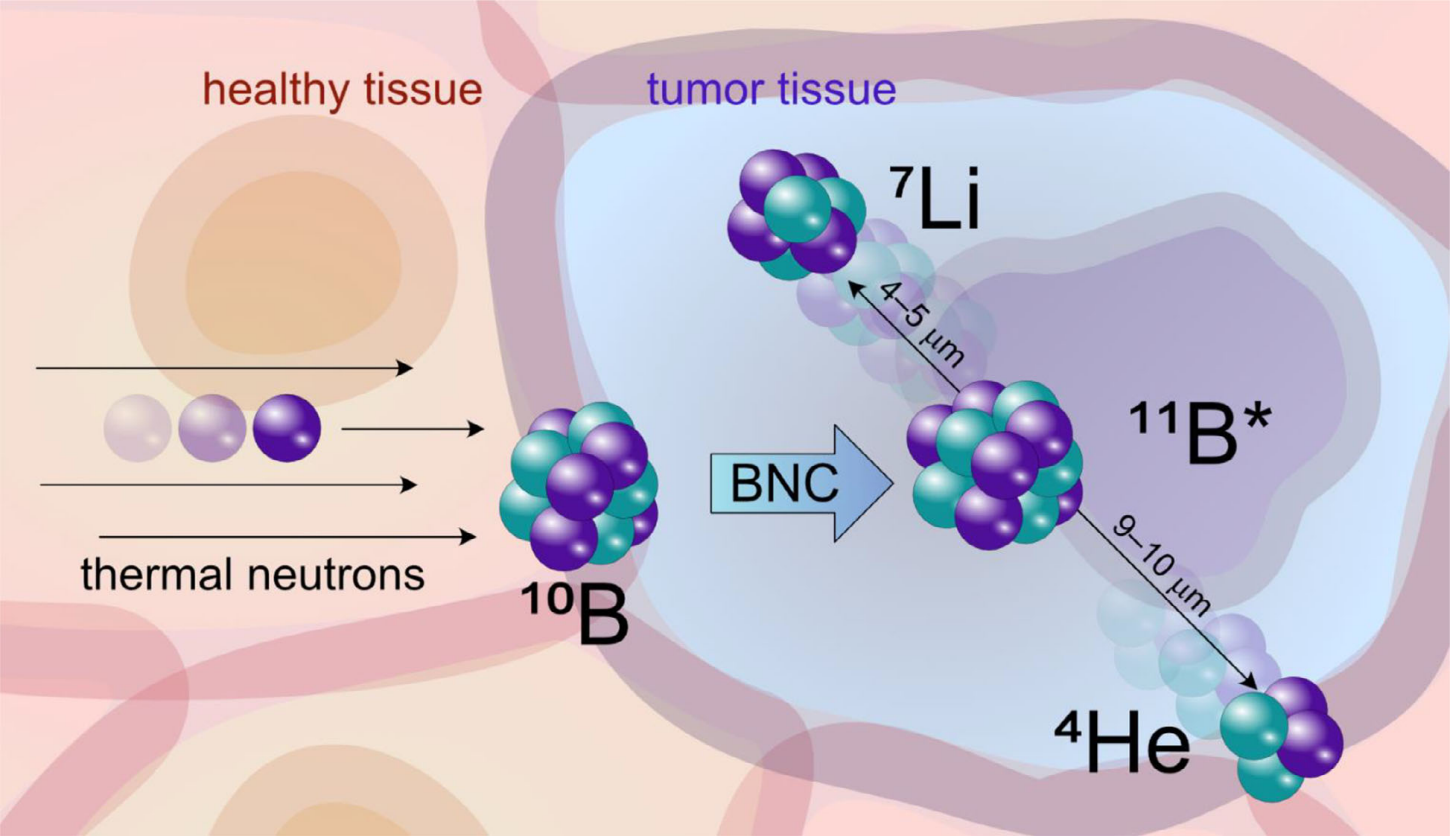
Simplified scheme of the boron neutron capture (BNC) reaction underlying BNCT: A thermal neutron is captured by a ^10^B nucleus, forming an excited ^11^B* intermediate that fissions into an α‐particle (^4^He) and a lithium nucleus (^7^Li), releasing ∼2.3 MeV of energy. The high‐LET particles travel only about one cell diameter (4–10 µm), confining the radiation damage to boron‐loaded tumor cells.

Cancer remains a major global health challenge, with limited options for recurrent, inoperable, or treatment‐resistant tumors [1, 2]. Boron neutron capture therapy (BNCT) has long been recognized as a promising strategy because it couples molecular targeting with highly localized radiobiological effects [3, 4, 5]. BNCT is a binary therapeutic approach that combines targeted drug delivery with nuclear physics. In the first step, tumor‐selective compounds enriched in ^10^B are administered to the patient. In the second step, the tumor region is exposed to low‐energy (thermal or epithermal) neutrons. When a neutron is captured by a ^10^B nucleus, an unstable ^11^B^*^ intermediate is formed, which rapidly undergoes fission to yield an α‐particle (^4^He) and a ^7^Li nucleus, together releasing ∼2.3 MeV of kinetic energy accompanied by weak γ‐radiation. Both, the α‐particle and the lithium ion exhibit very high linear energy transfer (LET), depositing dense ionization tracks along their ∼4–10 µm path length which is approximately the diameter of a single cell (Figure 1). This localized energy deposition produces clustered DNA damage that is difficult to repair, thereby achieving potent cytotoxicity within boron‐loaded cells while sparing neighboring healthy tissue [6, 7]. The physical and biological principles of BNCT, including neutron capture reactions, radiobiological effects, and dosimetric considerations, have been comprehensively described in recent reviews and monographs [5, 8, 9, 10, 11]. As the mechanism of BNCT is not the focus of this article, readers are referred to these sources for in‐depth discussions.

**FIGURE 2:**
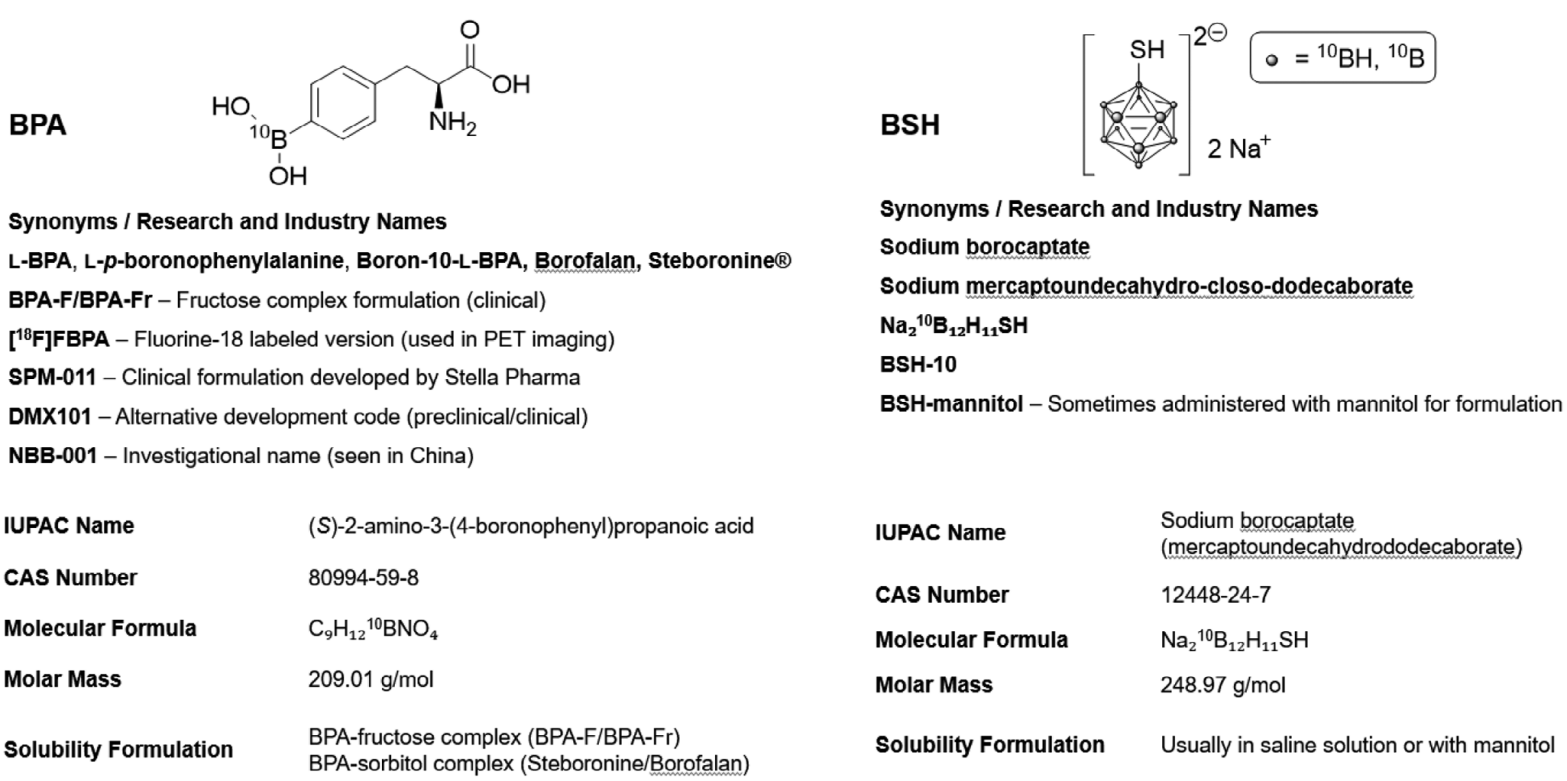
Overview and chemical and pharmacological data of the currently available compounds for BNCT, BPA and BSH.

The clinical trajectory of BNCT has been comprehensively reviewed in several recent publications. zhou et al. surveyed more than sixty clinical studies across glioblastoma and other CNS tumors, head and neck cancers, skin cancers, as well as smaller series in liver cancer and extramammary Paget's disease, with emerging trials targeting breast and pulmonary tumors. While BNCT is already approved for clinical use in Japan and selected regions, it was emphasized that nearly all registered trials remained in phase I/II, underscoring the urgent need for more phase II/III studies to rigorously define efficacy and safety [12].

barth et al. provided a historical and pharmacological perspective, revisiting the pioneering clinical use of sodium borocaptate (BSH, Na_2_B_12_H_11_SH, Figure 2) by hatanaka and 4‐borono‐l‐phenylalanine (l‐BPA, BPA, C_9_H_12_BNO_4_, Figure 2) by mishima. This review underscored that despite decades of development and intense research into novel boron delivery agents, only BSH and BPA have ever been evaluated clinically, with BPA emerging as the dominant agent for brain tumors and recurrent head and neck cancers [13].

shen et al. added a clinician's view, critically comparing responses and toxicities across trials in head and neck cancer, glioblastoma, melanoma, meningioma, breast cancer, and liver tumors. They identified significant variability linked to treatment protocols and patient factors and stressed the importance of standardized trial designs to allow robust comparison [14].

Finally, zhou et al. emphasized BNCT's potential as a “cell knife” and its growing integration into multimodal oncology strategies, including proton and carbon ion radiotherapy, ultrasound, and immunotherapy. While optimistic about synergistic applications, this review similarly acknowledged that clinical adoption remains limited by trial immaturity, reliance on just two boron agents, and unresolved issues in dose planning and boron pharmacokinetics [15].

Taken together, recent reviews highlight both the promise and the limitations of BNCT: clinical trials confirm safety and potential efficacy, yet all studies to date rely exclusively on the legacy agents BPA and BSH. This dependence reflects historical availability and regulatory familiarity but contrasts sharply with the rapid expansion of boron chemistry, which has produced numerous compounds with improved selectivity, multifunctionality, and theranostic potential [16, 17, 18, 19, 20, 21, 22, 23, 24]. None, however, have progressed to clinical testing [12]. This review therefore shifts the focus from trial expansion alone to the chemistry of next‐generation boron delivery agents and the translational challenges that must be overcome to move them beyond the laboratory.

## 2 The Clinical Reality: BSH and BPA

### 2.1 Chemical and Pharmacological Overview

The clinical history of BNCT has been defined almost exclusively by two boron delivery agents: BSH and BPA (Figure 2) [25]. Both compounds are considered the second generation of boron delivery agents (BDA) and were introduced more than half a century ago (BPA in 1958 and BSH in 1968) and, despite their shortcomings, remain the only agents to have been tested in patients [26, 27].

BPA is a phenylalanine analogue in which a *para*‐boronic acid substituent replaces the *para*‐hydrogen atom of the aromatic ring. Its tumor selectivity derives from uptake via the l‐type amino acid transporter 1 (LAT1), which is overexpressed in many malignancies, including glioblastomas and head and neck cancers [28, 29]. BPA in particular suffers from several physicochemical and pharmacological drawbacks. It has low boron content and very poor water solubility (0.6–0.7 g L^−1^ under neutral conditions), necessitating administration as a d‐fructose (BPA‐F or BPA‐Fr) or d‐sorbitol complex [30, 31, 32, 33]. The most widely used clinical formulation is Borofalan (^10^B), marketed in Japan as Steboronine, which received regulatory approval together with an accelerator‐based BNCT device (NeuCure and the dose calculation program NeuCure Dose Engine) in 2020 [34]. Other synonyms appearing in the literature include SPM‐011 (Stella Pharma's clinical‐grade BPA product), DMX101 (alternative development code, e.g., clinical trial NCT05737212), and NBB‐001 (e.g., in the Xiamen Humanity Hospital trial ChiCTR2200066473) [35, 36].

PET imaging with [^18^F]‐fluoroboronophenylalanine (^18^F‐FBPA, also known as NBB‐002) has been employed to predict tumor uptake and guide treatment planning [36, 37, 38]. Despite its utility, BPA suffers from relatively modest tumor selectivity and heterogeneous intratumoral distribution, which limit therapeutic margins [13, 15].

BSH is a dodecaborate cluster containing twelve boron atoms per molecule. Unlike BPA, it lacks an active transport mechanism and enters tumors primarily by passive diffusion or via regions of compromised blood‐brain barrier (BBB) [25]. This property underpinned its early use for glioblastoma, where barrier disruption is common [39]. It has often been administered as a sodium salt solution without the need for complexation. Pharmacokinetic analyses have revealed low tumor‐to‐normal‐tissue‐ (T/N) and tumor‐to‐blood‐ (T/B) ratios, reflecting poor selectivity, and accumulation in normal brain tissue is minimal in the absence of BBB disruption [13, 25]. While BSH offers high boron density per molecule, its limited tumor penetration and retention have curtailed its clinical utility.

Both agents do not reliably reach the ideal boron delivery threshold in all tumor types: clinical and preclinical studies often cite a target of ∼10^910^B atoms per cell (∼ 20–50 µg ^10^B /g tumor tissue) for effective BNCT, yet many tumors show lower accumulation [13, 31, 40]. BPA provides better T/N selectivity but is restricted by solubility issues and dependence on LAT1 expression, which varies between tumors. BSH, although highly boron‐dense, shows poor active targeting and relies on disrupted physiological barriers for uptake. In addition, both compounds have relatively rapid clearance, requiring continuous infusion during neutron irradiation, and their toxicity profiles, while generally manageable, leave limited room for dose escalation [40, 41].

Taken together, BPA and BSH represent historical solutions that enabled the clinical exploration of BNCT but do not meet the stringent requirements expected for modern targeted therapeutics. Their dominance in clinical trials reflects regulatory familiarity and accumulated safety data rather than optimal pharmacological performance.

### 2.2 Clinical Use Context

Despite decades of investigation, no boron delivery agent other than BSH and BPA has ever been administered in a human BNCT trial. All ongoing and completed clinical studies worldwide have relied on one (or both) of these two compounds. BPA is now the dominant agent for glioblastoma, head and neck cancer, and melanoma. BSH is used less frequently, largely restricted to brain tumors where a disrupted BBB facilitates passive uptake. This situation persists despite a rapidly expanding chemical literature reporting dozens of promising boron carriers with improved selectivity and multifunctionality [10, 12, 13, 14, 23, 42].

For nonclinical readers: phase I trials primarily assess safety and pharmacokinetics in small patient cohorts (often 10–30 patients), phase II trials evaluate preliminary efficacy and optimal dosing (tens to a few hundred patients), and phase III trials are large randomized studies confirming efficacy and safety before regulatory approval and are required to demonstrate clinical benefit against standard of care before regulatory approval.

To date, BNCT has not advanced beyond phase II in any cancer indication. This means that, although multiple small studies have reported encouraging tumor responses, the field lacks the robust phase III evidence that would allow widespread clinical adoption outside of Japan. Equally striking is the fact that the entire global BNCT clinical experience is based solely on BPA and BSH, underscoring the disconnect between chemical innovation and clinical translation.

### 2.3 Why only BPA and BSH?

The persistence of BPA and BSH in clinical BNCT trials is rooted in historical and logistical reasons rather than pharmacological superiority. Their early introduction in the 1960s and 1970s coincided with the establishment of the first hospital‐based BNCT programs, where both compounds were extensively characterized in terms of safety, dosimetry, and biodistribution [13]. At that time, neutron irradiation could only be performed at nuclear reactors, making clinical research highly site‐specific and limiting access for investigators developing alternative compounds [11]. This dependence on a few dedicated centers led to the accumulation of clinical expertise, technical protocols, and regulatory documentation specifically for BPA and BSH, creating a self‐reinforcing cycle of use. Even with the emergence of accelerator‐based neutron sources that have removed many infrastructural barriers, treatment planning, dosimetry, and pharmacokinetic models remain calibrated for these two agents, perpetuating their clinical dominance [11]. The extensive toxicological and pharmacokinetic data available for BPA and BSH have provided a uniquely strong regulatory foundation, facilitating continued approval and clinical deployment [43, 44].

Their well‐documented safety profiles enabled national regulatory acceptance, as exemplified by the approval of Borofalan (^10^B) for head and neck cancer treatment under Japan's national health insurance system in 2020 [5, 44, 45]. In contrast, new boron delivery agents would require full preclinical toxicology, isotopic production under GMP conditions, and early‐phase clinical evaluation, representing a considerable financial and administrative burden. The multi‐component nature of BNCT, which combines a pharmaceutical, a neutron source, and a radiotherapeutic workflow, further complicates regulatory pathways [14]. As a result, optimization has concentrated on formulation‐level advances, including monosaccharide or poly(vinylalcohol) complexes, and on combinatorial strategies such as the simultaneous administration of BPA and BSH or transient BBB disruption by d‐mannitol infusion or focused ultrasound [43, 46]. These incremental modifications have been viewed as more feasible than the clinical introduction of entirely new agents.

The translation of novel boron carriers into clinical testing is hindered by economic burdens and the limited commercial scale of BNCT. The field remains confined to a small number of specialized centers, reducing the potential market for any new agent. Preclinical development demands isotopic enrichment, scalable synthesis, and toxicological validation, each associated with high costs and limited funding opportunities [11]. Without industrial partnership or public‐sector investment, such development is rarely sustainable. Pharmaceutical efforts have therefore concentrated on maintaining production of clinical‐grade BPA formulations, such as Borofalan (^10^B) and its regional analogues, rather than advancing structurally novel candidates. Moreover, the absence of standardized international trial networks and harmonized dosimetric protocols has made multicenter validation of new agents logistically difficult [13, 14]. Collectively, these scientific, regulatory, and economic constraints explain why, despite extensive preclinical innovation in boron chemistry, only BPA, BSH, (and their formulations) have entered human trials.

The current clinical dependence on BPA and BSH therefore reflects historical precedent, regulatory conservatism, and limited economic incentives rather than a lack of chemical innovation. The following section examines the diverse range of boron delivery agents developed since 2018, highlighting how advances in molecular design, targeting strategies, and nanotechnology aim to overcome the pharmacological and translational limitations of these legacy compounds [16, 17, 18, 19, 20, 21, 22, 23, 24].

## 3 Emerging Boron Delivery Agents

### 3.1 Small Molecules

#### 3.1.1 Amino Acids

**SCHEME 1:**
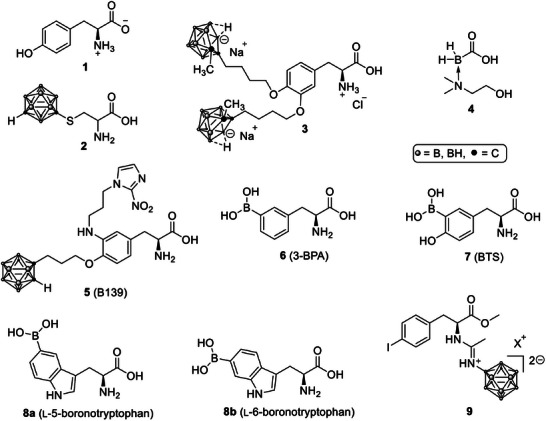
Boron‐containing amino acid derivatives for BNCT [24, 25, 26, 27, 28, 29, 30, 31].

Following clinical experience with BPA, considerable effort has been devoted to designing amino‐acid‐based boron carriers that retain transporter‐mediated uptake while overcoming BPA's poor solubility and metabolic instability. Small‐molecule derivatives remain attractive because of their predictable pharmacokinetics and compatibility with endogenous amino‐acid transport systems. In this context, several studies since 2018 have explored structural modifications aimed at enhancing boron content, stability, and tumor selectivity.

li et al. introduced fluoroboronotyrosine (FBY) as a metabolically stable BPA analogue that replaces the boronic acid with a trifluoroborate group, improving resistance to oxidative deboronation and allowing direct PET quantification of boron distribution through its ^18^F‐labelled form [47]. FBY (Scheme 1, **1**) showed LAT1‐dependent uptake in B16‐F10 melanoma cells, T/B‐ and tumor‐to‐muscle‐ (T/M) ratios above 3, and effective tumor control in a mouse model after neutron irradiation, with minimal systemic toxicity.

He et al. synthesized an α‐amino acid in which the cysteine side chain was substituted by a *meta*‐carborane cluster (**2**) [48]. This compound achieved picogram‐level intracellular boron accumulation in U87 glioma cells and induced marked cytotoxicity upon neutron exposure, even at fluences several orders of magnitude lower than those used for BPA. Interestingly, modest cytostatic activity was observed even without irradiation, suggesting a potential chemo‐radiotherapeutic synergy.

Building on the concept of coupling boron clusters to aromatic or catechol‐type backbones, zhu et al. conjugated *nido*‐carborane to l‐DOPA (**3**), yielding a highly water‐soluble carboranyl levodopa derivative with significant in vitro antitumor efficacy against C6 glioma cells [49]. This strategy demonstrates that carborane modification can preserve amino‐acid transportability while greatly enhancing boron payload and solubility.

zhu et al. reported carboxyboranylamino ethanol (**4**), a small boron molecule that achieved T/B‐ and T/N‐ratios (brain tissue) of 2.9 and 27.8, respectively, with in‐vitro tumor cell killing more than 50‐fold greater than that of BPA [50]. Molecular‐docking analysis suggested strong interactions with epidermal growth factor receptor (EGFR) and c‐MYC, possibly accounting for enhanced intracellular delivery and antitumor potency.

Efforts to retain transporter‐mediated uptake while improving physicochemical liabilities have produced several BPA‐inspired designs. A dual‐target phenylalanine series that couples LAT1 recognition with hypoxia activation via a nitroimidazole trigger achieved selective accumulation in hypoxic regions and prolonged intratumoral retention; lead B139 (**5**) reached peak tumor boron of ∼50 µg g^−^^1^ with T/B > 3 in vivo and showed acceptable acute and subacute toxicity in mice, indicating a feasible therapeutic window for BNCT scheduling [51].

A positional‐isomer strategy has been proposed to mitigate BPA's solubility constraint. 3‐BPA (**6**) exhibited water solubility of ∼125 g L^−1^ at neutral pH (compared to 0.6–0.7 g L^−1^ for *para*‐substituted BPA), eliminating the need for fructose or sorbitol while maintaining LAT1‐dependent uptake and BPA‐like biodistribution in vitro and in vivo. These results suggest that 3‐BPA could function as a drop‐in replacement for 4‐BPA with simplified formulation and reduced excipient burden, pending clinical verification [32].

Several recent studies have sought to retain LAT1‐mediated transport while improving solubility, dosing flexibility, and intratumoral persistence. A borylated amino acid analogue of l‐tyrosine, 3‐borono‐l‐tyrosine (BTS, **7**), was reported with higher in‐vitro uptake and retention, reduced competition by natural amino acids, and the ability to be formulated and administered as a bolus at higher levels than BPA, yielding two to three times greater boron delivery in vivo with favorable T/B ratios; uptake correlated with LAT1 expression, indicating a transporter‐anchored mechanism with potentially improved practicality for clinical scheduling [52].

Beyond phenylalanine analogues, tryptophan‐based boronoamino acids were introduced as alternative LAT1‐addressed scaffolds; enantiopure 5‐ and 6‐boronotryptophans (e.g., **8a** and **b**) showed cell‐line‐dependent uptake with molecular‐dynamics support for transporter compatibility and were proposed as candidates in settings where BPA transport is suboptimal, aligning with a precision‐medicine rationale for transporter‐guided agent selection [53]. In parallel, vectorized *closo*‐dodecaborate constructs bearing an iodoaromatic handle (**9**) were described to enable tracking by X‐ray computed tomography. Preclinical biodistribution and contrast measurements suggested the feasibility for imaging‐assisted assessment of organ distribution and dose planning, with dose‐finding and optimization of iodine density identified as next steps [54].

A broader perspective on boronated amino acids has recently been published, focusing on synthetic strategies, structure‐activity relationships (SAR), transporter interactions, and common liabilities such as poor solubility and rapid elimination. [18] That review is complementary to the present work, which concentrates on the most recent agents and data and on their translational implications within the wider BNCT pipeline.

Taken together, these reports illustrate two converging directions: incremental isomeric and scaffold refinements that solve practical formulation and dosing constraints while preserving LAT1 targeting [32, 52], and alternative amino‐acid frameworks or vectorized cluster designs that expand transporter options and add imaging capability for selection and planning [53, 54].

#### 3.1.2 Nucleic Acids

Efforts to integrate boron clusters into nucleic‐acid scaffolds have expanded beyond the purine and pyrimidine conjugates mentioned in earlier literature [5]. Recent research has focused on boron‐substituted nucleosides, antisense constructs, and aptamers, reflecting a growing interest in combining gene‐targeting or recognition capabilities with boron delivery.

**SCHEME 2:**
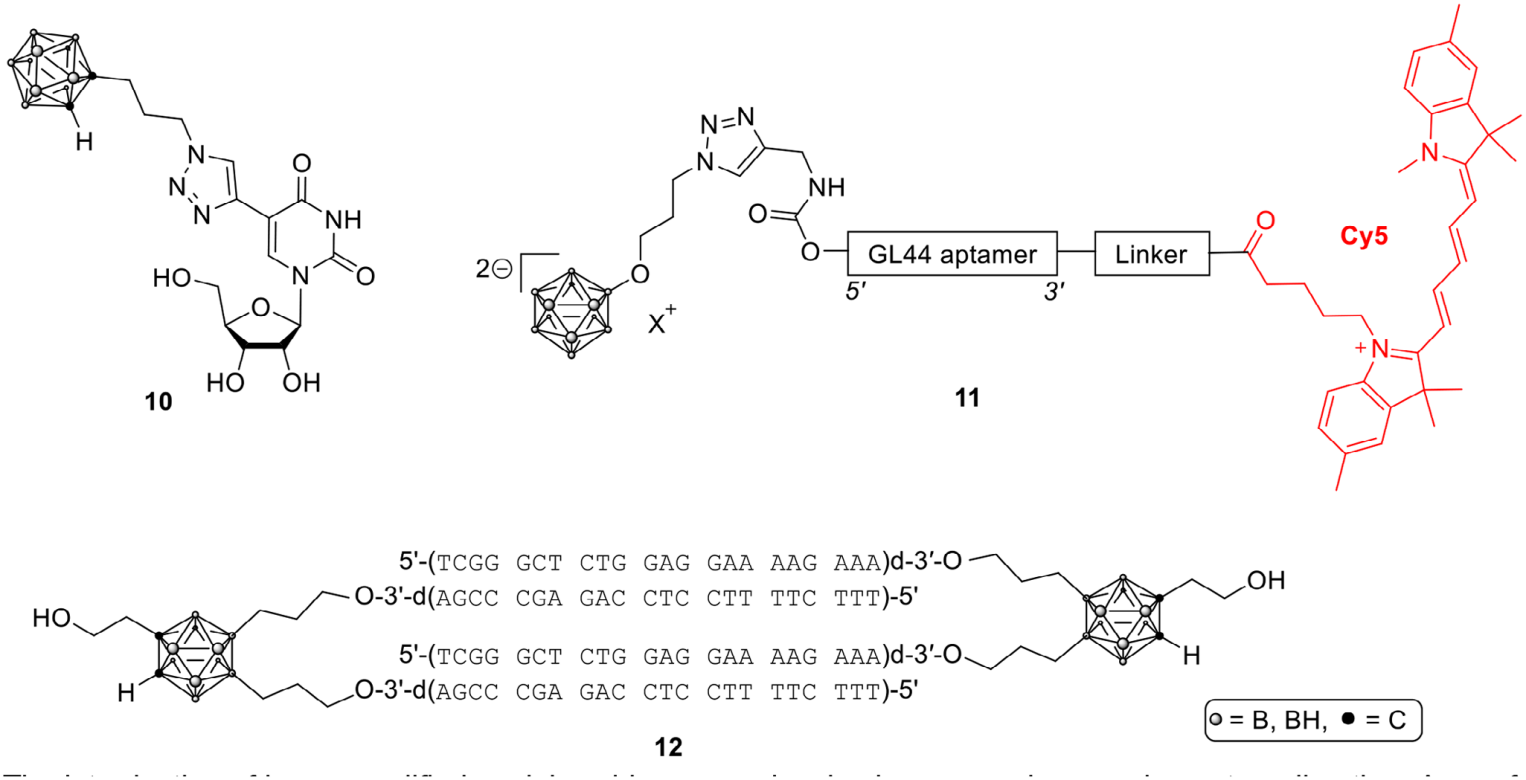
Boron‐containing nucleic acid derivatives for BNCT [32, 33, 34, 35].

A series of uridine and 2′‐deoxyuridine conjugates (e.g., Scheme 2, **10**) bearing *ortho*‐carborane clusters was prepared via sonogashira coupling and husigen‐meldal‐sharpless “click reaction,” achieving low to moderate cytotoxicity and efficient phosphorylation by deoxycytidine kinase (dCK). Their susceptibility to enzymatic activation together with favorable tolerability identified these conjugates as promising small‐molecule carriers capable of intracellular trapping through nucleoside‐metabolic pathways [55]. Parallel developments in the field of charged‐particle therapy underscored that the accumulated knowledge from BNCT compound design could also guide optimization for boron proton‐capture enhanced proton therapy (BPCEPT), suggesting that nucleobase‐linked and cluster‐based scaffolds may be transferable between modalities with careful adjustment of isotope enrichment and pharmacokinetics [56].

The introduction of boron‐modified nucleic acid macromolecules has opened a complementary direction. A proof‐of‐concept study demonstrated that a *closo*‐dodecaborate‐tagged 2′‐F‐RNA (= nuclease‐resistant RNA oligonucleotides with all pyrimidine nucleotides substituted by 2’‐fluoro‐modified analogs) aptamer (GL44, **11**), selective for human glioblastoma U‐87 MG cells, achieved specific internalization and boron delivery sufficient to induce a viability reduction after neutron irradiation comparable to that of BPA [57]. The aptamer displayed low toxicity and potential for modular boron loading, providing a basis for future optimization through isotope enrichment and multivalent cluster attachment. Similarly, boron‐decorated antisense oligonucleotides (B‐ASOs) targeting EGFR were constructed from 1,2‐dicarba‐*closo*‐dodecaborane conjugates (**12**). These nanostructures formed toroidal assemblies with enhanced nuclease stability, cytoplasmic localization, and efficient gene‐silencing activity without triggering inflammasome activation, while also achieving measurable boron accumulation within tumor cells [58].

Collectively, these studies demonstrate the growing versatility of nucleic‐acid‐based carriers for BNCT, spanning metabolically activated nucleoside analogues to programmable oligonucleotide nanostructures [55, 57, 58]. Although still at an early experimental stage, such constructs combine molecular recognition, therapeutic synergy, and controllable boron loading, offering a distinct design space that complements traditional small‐molecule boron agents.

#### 3.1.3 Porphyrins and Analogs

Tetrapyrrolic scaffolds continue to attract interest as boron carriers because they combine intrinsic tumor affinity with photophysics amenable to theranostics and to dual photodynamic therapy (PDT)‐BNCT concepts. Recent work has focused on improving formulation, imaging guidance, and boron payload while maintaining biocompatibility.

**SCHEME 3:**
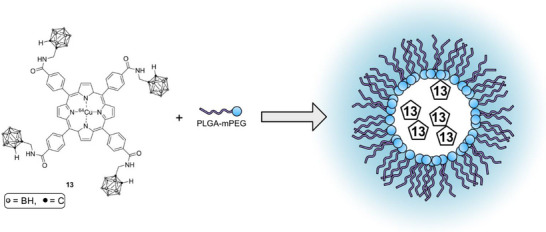
Synthesis and structure of the boronated porphyrin–PLGA‐mPEG nanomicelle (BPN) used for imaging‐guided BNCT [36].

An imaging‐guided strategy used a boronated porphyrin (Scheme 3, **13**) encapsulated in poly(lactide‐co‐glycolide)‐monomethoxy‐poly(polyethylene‐glycol) (PLGA‐mPEG) micelles to create a nanocomplex that reduced the hematologic toxicity reported for earlier porphyrins and enabled in vivo fluorescence and ^64^Cu PET quantification of boron. PET kinetics showed a multi‐dose schedule that raised tumor boron to ∼125 ppm with very high T/N ratios, and neutron irradiation produced near‐complete tumor suppression in a melanoma model, illustrating a practical theranostic workflow for BNCT planning and delivery [59].

**SCHEME 4:**
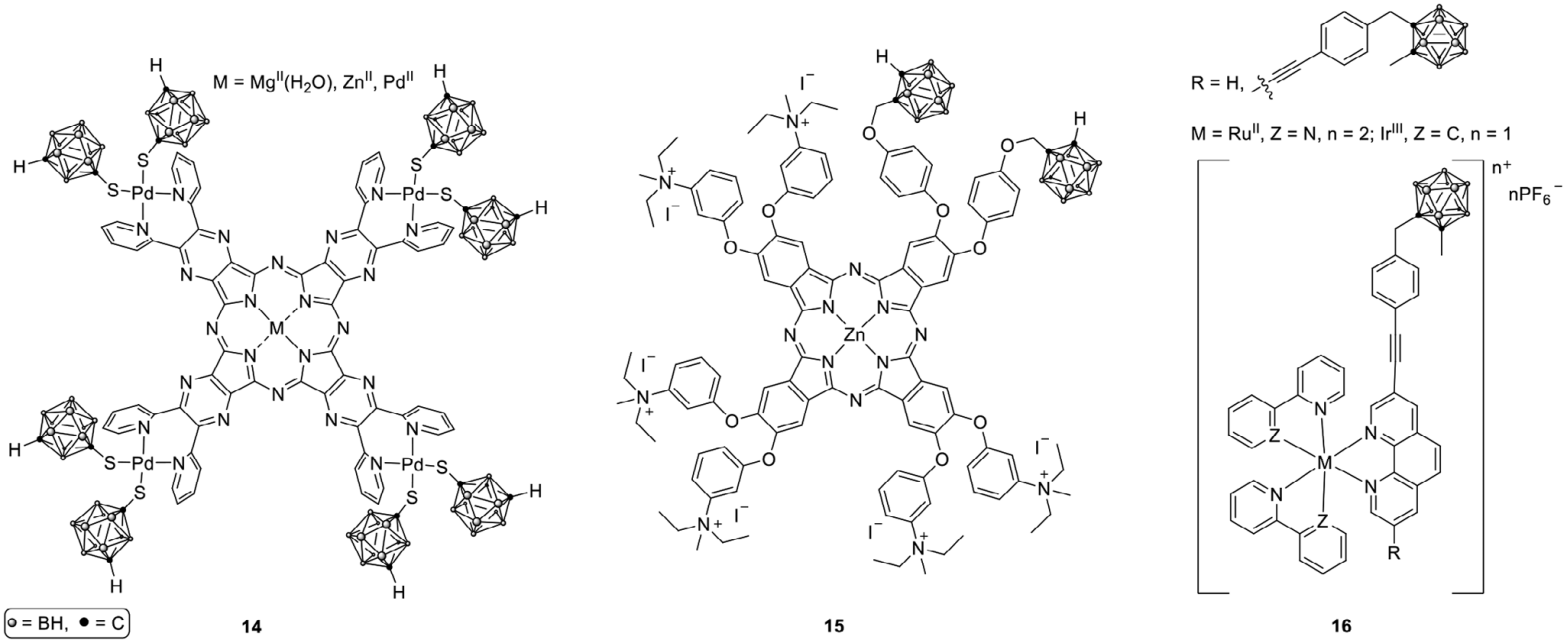
Structures of carborane‐loaded, porphyrazines, phthalocyanines, and phenanthrolines as potential boron carriers and photosensitizers in BNCT and PDT [37, 38, 39].

Macrocyclic platforms with high boron content have also progressed. Pentanuclear porphyrazines bearing eight external *meta*‐carboranethiolate groups (Scheme 4, **14**) were prepared and characterized; they generated singlet oxygen efficiently in solution and were proposed as bimodal PDT‐BNCT agents, with formulation into aqueous carriers suggested for biological evaluation [60].

In parallel, phthalocyanine‐carborane conjugates (**15**) were rendered water soluble via quaternization to give a hexacationic zinc(II) complex that delivered substantial boron to osteosarcoma cells at sub‐ppm exposure while maintaining mild dark toxicity, again supporting the feasibility of tetrapyrrole frameworks as high‐payload carriers [61].

A complementary line of work appended carborane units to Ru^II^ and Ir^III^ phenanthroline photosensitizers, yielding boron‐rich complexes (**16**) that showed good cellular internalization and high singlet‐oxygen quantum yields under irradiation while lacking dark toxicity under PDT culture conditions. Although evaluated primarily for PDT, their boron content and cell uptake identify them as potential dual‐use candidates and as modular components for future BNCT‐oriented designs [62].

Across porphyrins, porphyrazines, phthalocyanines, and metal‐carborane photosensitizers, the field is converging on theranostic, high‐payload, and formulation‐aware designs that can both quantify boron in real time and deliver it efficiently to tumors, providing practical levers to optimize BNCT scheduling and selectivity [59, 60, 61, 62].

#### 3.1.4 Carbohydrates

Many cancer cells show an increased rate of glycolysis and an overexpression of glucose transporters (GLUTs). This metabolic feature has encouraged the development of sugar‐based boron carriers that combine high water solubility with facilitated cellular uptake through GLUTs. In some cases, these compounds also include imaging functions that allow visualization of boron distribution in tumors [63]. Recent studies on carbohydrate‐based boron delivery agents can be grouped into three main directions: (i) sugar‐carborane conjugates with high boron content, (ii) glucose analogues that specifically target GLUT transporters, and (iii) carbohydrate‐based nanocarriers designed for dual therapeutic and imaging applications.

**SCHEME 5:**
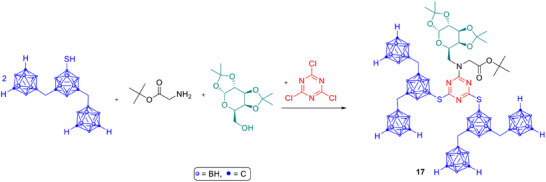
Synthesis strategy for a carbohydrate‐conjugated modular boroncarrier system based on 1,3,5‐triazine with up to 60 boron atoms per molecule.

*Sugar‐carborane conjugates with high boron content*: New synthetic approaches now make it possible to attach monosaccharides to highly boron‐loaded scaffolds. One example is a 1,3,5‐triazine‐based system bearing a galactopyranosyl unit (Scheme 5, **17**), which achieved around 60 boron atoms per molecule and serves as a flexible platform for coupling with tumor‐selective biomolecules [64, 65]. Another strategy involves the preparation of cyclic boronic acids at the anomeric position of sugars by hydroboration of open‐chain intermediates.

These compounds showed low toxicity in human fibroblast tests and provided a simple route toward stable boron‐sugar analogues, although tumor‐cell uptake has yet to be demonstrated [66].

**SCHEME 6:**
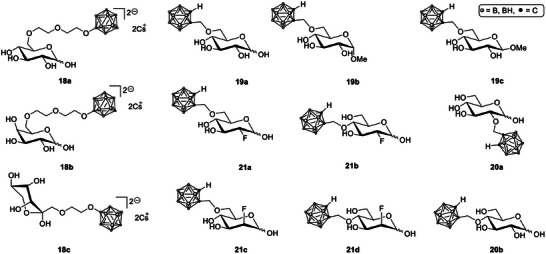
GLUT1‐targeted conjugates of carbohydrates and boron clusters for BNCT.

*GLUT‐targeted glucose analogues*: A comprehensive study of *ortho*‐carboranylmethyl glucose derivatives (Scheme 6, **18a–c**) mapped the positions on the glucose molecule that optimize GLUT1 binding, low toxicity, and efficient boron delivery. Several isomers surpassed the established agents BPA and BSH in cellular boron accumulation while avoiding interference with normal glucose metabolism. These findings strengthen the concept of using glucose as a “Trojan horse” for boron transport (**19a–c**, **20a**, **20b**) [67, 68]. Recent work by matović et al. further expanded the GLUT‐targeting concept by developing fluorinated carbohydrate conjugates (**21a–d**) designed to mimic clinically used imaging agents. Specifically, the authors synthesized fluorinated glucose (FDG, **21a** and **21b**) and fluorinated mannose (FDM, **21c** and **21d**) analogues, the nonradioactive counterparts of the well‐known PET tracers 2‐deoxy‐2‐[^18^F]fluoro‐d‐glucose and 2‐deoxy‐2‐[^18^F]fluoro‐d‐mannose, each bearing a carboranyl group. These compounds were evaluated for their cytotoxicity, affinity to glucose transporters, and boron delivery capacity in vitro.

One of the fluorinated mannose derivatives achieved the best balance between low toxicity, strong GLUT1 affinity, and high boron uptake. However, the study also revealed strong and prolonged binding of these glycoconjugates to plasma proteins, which could limit the amount of compound available for tumor uptake [69].

*Hyaluronan and carbohydrate‐based theranostic systems*: Hyaluronic acid (HA, Scheme 8, **22**) has been used to solubilize hydrophobic carboranes (**23**) while simultaneously enabling tumor targeting through CD44 (cell‐surface glycoprotein) receptors. An HA‐carborane complex showed efficient cellular uptake, visible subcellular localization through fluorescence, and BNCT activity that matched or exceeded the performance of BPA‐fructose [70]. A related HA‐based nanogel system (**24** and **25**) encapsulated a fluorescent aza‐BODIPY dye linked to ^10^B‐enriched BSH (**26**). This formulation achieved long tumor retention and improved T/N ratios in glioblastoma models, suggesting strong potential for image‐guided BNCT [71].

**SCHEME 7:**
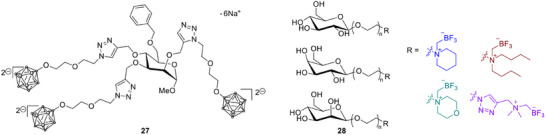
Low‐molecular‐weight α‐d‐mannopyranoside derivative (MMT1242) and potential sugar‐trifluoroborate conjugate theranostics for BNCT.

In another study, a low‐molecular‐weight α‐d‐mannopyranoside derivative (MMT1242, Scheme 7, **27**) showed broad intracellular distribution, longer tumor retention than BPA or BSH, and significant tumor growth inhibition in vivo. These results identify MMT1242 as a promising candidate for future clinical studies [72]. Finally, sugar‐trifluoroborate conjugates (**28**) were proposed as theranostic compounds suitable for PET imaging. Although one radiolabeled derivative was chemically stable and easily visualized in vivo, its toxicity at higher concentrations limits its direct use in BNCT, suggesting that such compounds may be better suited for diagnostic imaging rather than therapy [73].

**SCHEME 8:**
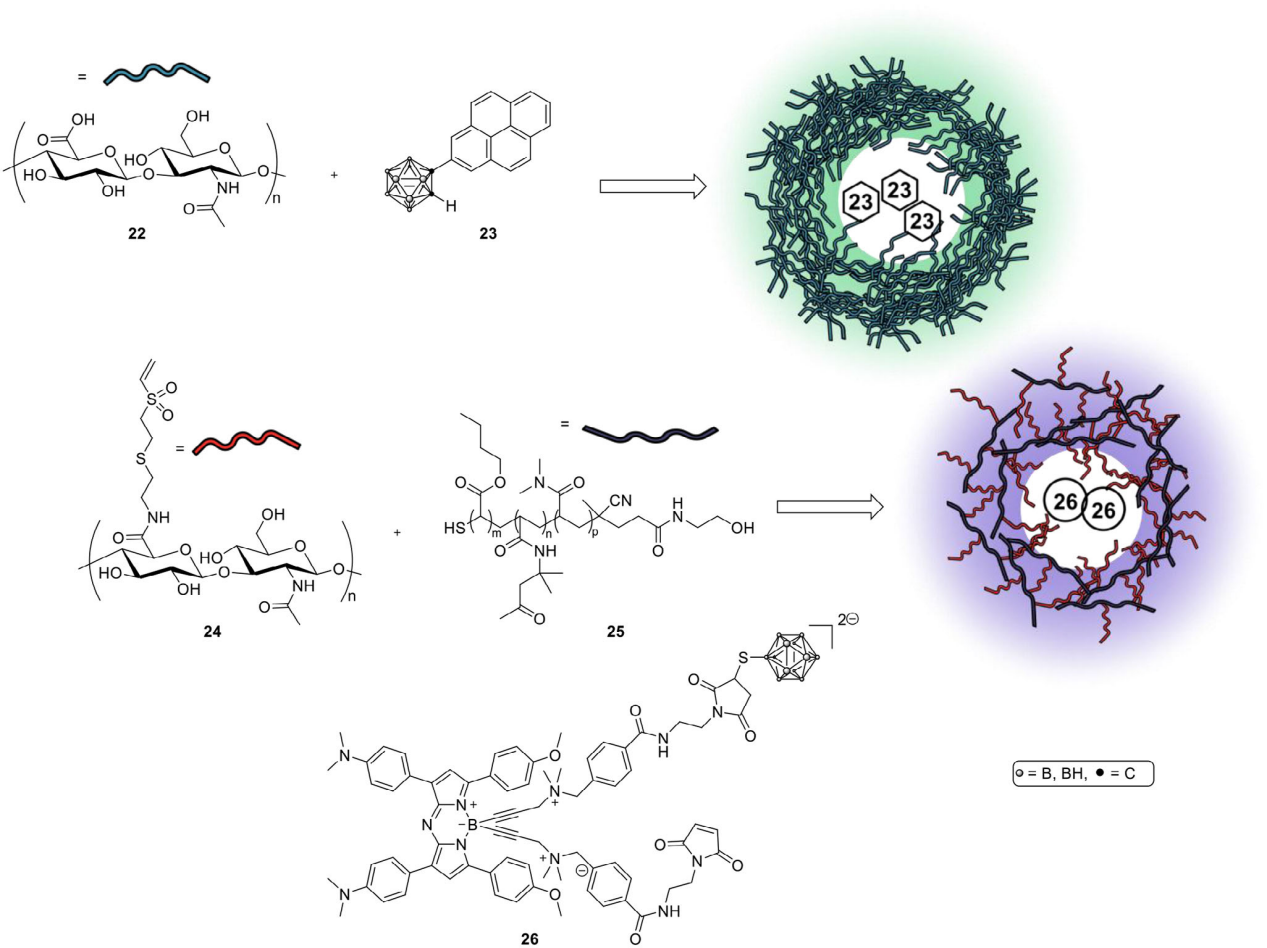
Complex of hyaluronic acid and a fluorescent carborane (top) and hyaluronic acid‐based nanogels as theranostic boron delivery systems for BNCT.

Recent advances in carbohydrate‐based boron carriers highlight two promising directions: selective uptake through overexpressed transporters such as GLUT1 or CD44, and the integration of imaging capabilities for treatment planning. Several of these new systems have achieved higher boron delivery to cancer cells than the traditional agents BPA and BSH. However, further progress will depend on improving their stability, pharmacokinetics, and safety profiles before moving toward clinical application [63, 69].

#### 3.1.5 Other Biomimetics

Besides amino acids, nucleic acids, porphyrins, and carbohydrates, several other biomimetic molecules have recently been explored as potential boron carriers for BNCT. These compounds are often inspired by biologically active structures that show inherent tumor selectivity or favorable pharmacokinetic behavior. Their modification with boron‐containing clusters has yielded a diverse set of molecules with both therapeutic and diagnostic potential.

**SCHEME 9:**
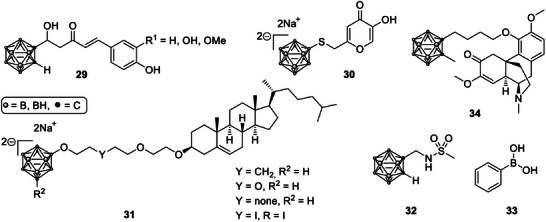
Structures of boron‐containing small molecule biomimetics for BNCT [49, 50, 51, 52, 53, 54].

A novel class of boronated curcumin derivatives, known as boronated monocarbonyl analogues of curcumin (BMACs, Scheme 9, **29**), was synthesized by azzi et al. [74]. In these compounds, an *ortho*‐carborane cage replaced one of the phenolic rings of curcumin, improving both antitumor activity and stability in aqueous solutions. The BMAC molecules displayed significant cytotoxic activity against cancer cell lines while maintaining the ability to inhibit β‐amyloid aggregation, suggesting their dual potential for oncology and neurodegenerative applications. The presence of boron within the carborane cage also makes these derivatives attractive candidates for BNCT, particularly if coupled with nanocarrier systems such as liposomes or cyclodextrins to enhance solubility and tumor targeting [74].

To improve selective boron accumulation in brain tumors, takeuchi et al. developed a new compound, kojic acid‐dodecaboranethiol (KA‐BSH, **30**). The conjugation of kojic acid, known for its affinity to certain cancer cell types, with the boron‐rich dodecaborate cluster resulted in a compound capable of achieving high T/B boron ratios in a rat glioma model. BNCT with KA‐BSH significantly prolonged survival in tumor‐bearing rats, indicating the promise of this molecule as a practical intravenous boron carrier for glioma treatment [75].

druzina et al. reported the synthesis of *closo*‐dodecaborate‐containing cholesterols (**31**), designed for incorporation into liposomal drug delivery systems. These boronated cholesterols can serve as membrane‐stabilizing components in liposomes, enabling the codelivery of boron with therapeutic agents directly to tumor sites [76].

A particularly innovative approach was presented by alberti et al., who designed a carborane‐containing inhibitor of carbonic anhydrase IX (CAIX, **32**), an enzyme overexpressed in several cancers, including mesothelioma and breast carcinoma. The compound, named CA‐SF, combines enzyme inhibition with boron delivery, producing synergistic cytotoxicity when followed by neutron irradiation. In vivo experiments demonstrated markedly reduced tumor regrowth in mice treated with CA‐SF and BNCT, showing how targeted molecular therapies can be successfully combined with neutron capture strategies [77].

More recently, chen et al. investigated phenylboronic acid (PBA, **33**) as a simple and effective nucleus‐targeting boron agent for BNCT. PBA exhibited good solubility, low toxicity, and a unique ability to accumulate within cell nuclei through importin‐mediated transport. Molecular docking studies confirmed strong binding to nuclear transport proteins, suggesting that PBA may be an efficient nuclear delivery agent for boron, particularly in melanoma cells, where enhanced nuclear localization could improve treatment outcomes [78]. A notable example of biomimetic boron agents is the conjugation of the natural alkaloid sinomenine with a carborane cluster to create methylcarboranyl‐*n*‐butyl sinomenine (**34**). This hybrid retained the biological framework of the traditional Chinese medicine while introducing a high boron payload and improved molecular interactions with matrix metalloproteinases (MMP‐1 and MMP‐13), which are overexpressed in inflammatory and tumor tissues. The compound showed higher membrane permeability and greater in vitro cytotoxicity toward both glioma and fibroblast‐like synoviocyte cells compared with BPA, suggesting dual potential for boron neutron capture synovectomy and BNCT [79].

Together, these studies demonstrate the versatility of boron chemistry in the design of novel biomimetic carriers. From plant‐derived natural products and enzyme inhibitors to lipid and nuclear‐targeting molecules, such approaches highlight how structural diversity can be leveraged to achieve improved tumor selectivity, better biodistribution, and enhanced therapeutic synergy with neutron irradiation.

#### 3.1.6 Boronated Molecular‐Targeted Drugs

The development of boron‐containing molecular‐targeted drugs has focused on exploiting receptors and transport systems that are overexpressed on the surface of tumor cells. By coupling boron clusters to ligands or inhibitors that naturally accumulate in cancerous tissues, researchers aim to achieve selective tumor delivery while minimizing systemic toxicity. Recent years have seen considerable progress in designing boron conjugates that engage targets such as the folate receptor, integrins, matrix metalloproteinases, and glucose transporters, among others.

**SCHEME 10:**
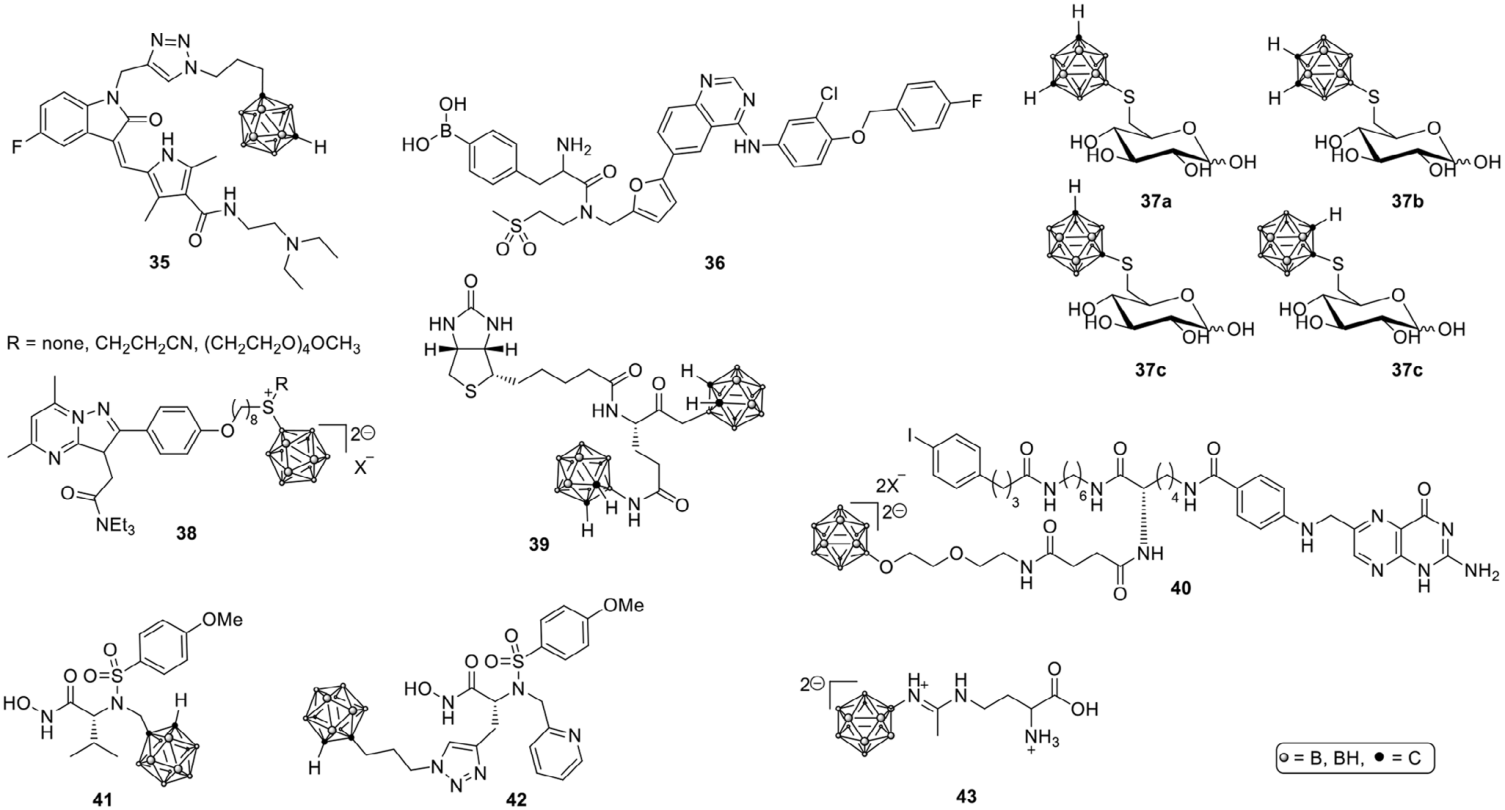
Structures of small molecules boronated molecular‐targeted drugs as potential boron carriers in BNCT [55, 56, 57, 58, 61, 62, 63, 64, 65, 66, 67].

A hybrid strategy combining chemotherapy and BNCT was presented by couto et al., who synthesized sunitinib‐carborane conjugates designed to inhibit tyrosine kinase receptors that are overexpressed in gliomas. One hybrid compound (Scheme 10, **35**) exhibited strong cytotoxicity against glioma cells and proved to be substantially more effective than both sunitinib and BPA‐fructose in neutron irradiation experiments. The study demonstrated the potential of dual‐action molecules that unite targeted inhibition and boron delivery within a single pharmacophore [80]. Following the sunitinib‐carborane hybrids that showed enhanced cytotoxicity and neutron‐dependent killing in glioma cells, a related approach was reported with a lapatinib‐boronophenylalanine conjugate (Lap‐BPA, **36**) [81]. This compound combined the targeting capacity of the epidermal growth factor receptor inhibitor lapatinib with the boron delivery function of BPA. Lap‐BPA exhibited over sixfold higher solubility than BPA, maintained low cytotoxicity, and achieved selective uptake in tumor cells with high intracellular boron levels. The design demonstrates that coupling small‐molecule kinase inhibitors to BPA can enhance tumor selectivity and pharmacological handling, offering a promising avenue for the next generation of targeted BNCT agents [81].

Targeting other cancer‐specific transport systems, matović et al. explored a series of 6‐deoxy‐6‐thio‐carboranyl‐d‐glucoconjugates (**37a**‐**d**) aimed at the GLUT1 transporter. The study revealed that even minor structural variations in the boron cluster or linker strongly influenced GLUT1 affinity and boron delivery capacity. Although one compound displayed fivefold higher boron uptake compared to BPA and BSH, others acted as GLUT1 antagonists rather than carriers. The findings emphasized the delicate structural balance required for effective transporter‐mediated BNCT targeting [82].

The translocator protein (TSPO), which is overexpressed in several cancer types, has also been investigated as a BNCT target. hattori et al. synthesized a series of dodecaborate‐containing pyrazolopyrimidines (**38**) designed to bind TSPO. Among these, DPA‐BSTPG showed particularly high boron delivery and water solubility, as well as efficient tumor cell killing after neutron irradiation in vitro. In vivo evaluation of this compound is currently underway [83].

**SCHEME 11:**
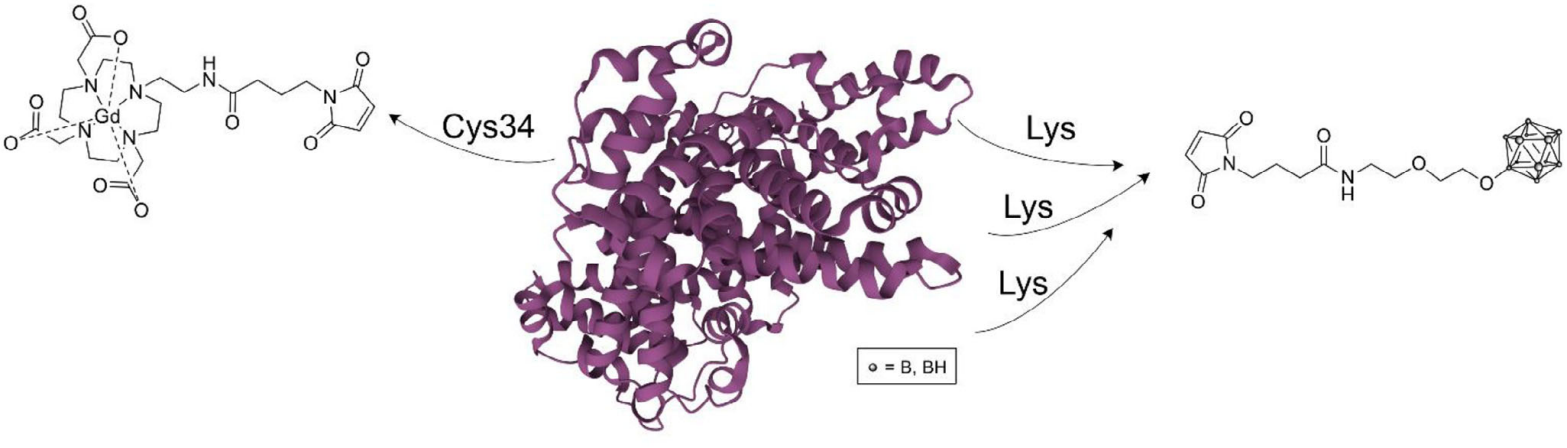
Chemical structure and synthetic scheme of integrin‐α_v_β_3_ targeted long‐retention‐type boron carrier cRGD‐MID‐AC [59]. (Image based on PDB 3V03) [60].

Integrin α_v_β_3_, a key mediator of tumor invasion and angiogenesis, has emerged as another attractive target. tsujino et al. developed a cyclic RGD‐functionalized *closo*‐dodecaborate‐albumin conjugate (cRGD‐MID‐AC, Scheme 11) that exploits both albumin‐based delivery and integrin targeting. In glioma‐bearing rats, this conjugate achieved high tumor accumulation and significant therapeutic effects after BNCT, leading to long‐term survival in some animals. Because its uptake mechanism differs from that of BPA, the compound also holds promise for combination therapy involving multiple boron carriers [84].

Similarly, gruzdev et al. synthesized a series of d‐biotin conjugates containing *closo*‐ and *nido*‐carborane residues (e.g., Scheme 10, **39**, Image based on PDB 3V03) [85]. Biotin is recognized by cancer cells through the sodium‐dependent multivitamin transporter, which can enhance tumor targeting. The authors prepared several variants, including a biotin conjugate with two carborane cages and a glutamic acid residue, laying the groundwork for further biological evaluation as selective boron delivery agents [86].

A more complex, multifunctional design was reported by nishimura et al., who developed PBC‐IP (**40**), a boron compound integrating a folate receptor‐targeting moiety, a boron‐rich core, and an albumin‐binding domain. PBC‐IP exhibited low toxicity, good water solubility, and high tumor accumulation after both intravenous and convection‐enhanced administration. When used in cozmbination with BPA, it produced remarkable survival benefits in glioma‐bearing rats, with some animals remaining tumor‐free for six months after BNCT [87].

Enzyme‐targeted strategies have also emerged as a promising approach. flieger et al. reported the synthesis of carborane‐bearing hydroxamate inhibitors of matrix metalloproteinases (MMP‐2, MMP‐9, and MMP‐13), enzymes that play crucial roles in tumor invasion. These inhibitors (**41** and **42**) displayed nanomolar potency and strong tumoricidal effects after neutron irradiation, achieving several‐fold higher BNCT efficacy than BPA in vitro. Notably, their therapeutic action was attributed to high‐affinity binding to extracellular MMPs rather than cellular internalization, introducing a new concept of surface‐targeted BNCT [88].

Finally, ryabchikova et al. synthesized *closo*‐dodecaborate‐based amidines that mimic amino acid structures to target the LAT1 transporter. One compound (**43**) demonstrated low toxicity, good solubility, and selective accumulation in experimental melanoma models, with T/N ratios exceeding 6. These results identify it as a promising candidate for further preclinical BNCT evaluation [89].

Recent studies have explored the incorporation of boron clusters into agents that interact with growth factor pathways, particularly the epidermal growth factor receptor (EGFR), to achieve tumor‐selective delivery and dual molecular‐radiation therapy for BNCT. One strategy employs boron‐decorated antisense oligonucleotides (B‐ASOs) designed to downregulate EGFR expression while serving as boron carriers. These constructs, functionalized with metallacarborane clusters such as the ferrabis(dicarbollide)‐anion (FESAN), were shown to enter cancer cells via receptor‐mediated endocytosis without the need for transfection agents. In EGFR‐overexpressing A431 and U87‐MG cells, the antisense activity effectively suppressed EGFR expression and achieved T/N ratios around 4.8, highlighting the feasibility of receptor‐guided nucleic acid delivery for BNCT [90].

Complementary progress has been made in small‐molecule EGFR inhibitors incorporating boron clusters. alamón et al. developed a hybrid compound combining a carborane core with the 4‐anilinoquinazoline scaffold of erlotinib. The hybrid selectively accumulated in glioma cells, exhibited superior in vitro BNCT efficacy compared with BPA, and retained intrinsic antitumor activity in U87‐MG glioblastoma models. Liposomal encapsulation further enhanced brain delivery, yielding significant tumor suppression and extended survival [91].

Building on this concept, dávila et al. introduced a new generation of 3D carborane‐based bioisosteres of erlotinib to improve physicochemical and pharmacological profiles [92]. Several derivatives displayed markedly greater cytotoxicity (up to >12‐fold vs. erlotinib) and up to sevenfold selectivity for glioblastoma over astrocytes. The lead compound moderately inhibited both wild‐type and mutant EGFR^T790M^, triggered apoptosis as the primary cell‐death pathway, and showed excellent in vivo safety and predicted blood‐brain barrier permeability. These results underscore the potential of carborane substitution to enhance both boron payload and therapeutic performance while maintaining drug‐like properties.

Taken together, these studies illustrate the rapid diversification of molecular‐targeted boron carriers. By incorporating boron clusters into ligands for defined receptors, enzymes, and transporters, researchers are building a new generation of agents that combine molecular precision with therapeutic potency. The most advanced examples, such as PBC‐IP and cRGD‐MID‐AC (Scheme 11), achieve both strong tumor selectivity and sustained boron retention, moving BNCT closer to the standards of contemporary targeted cancer therapy. In parallel, EGFR‐directed nucleic acids and small‐molecule boron hybrids delineate a coherent framework for dual‐function therapeutics that unite molecular targeting with BNCT‐mediated cytotoxicity.

#### 3.1.7 Mitochondria‐ and DNA‐Targeted Drugs

**SCHEME 12:**
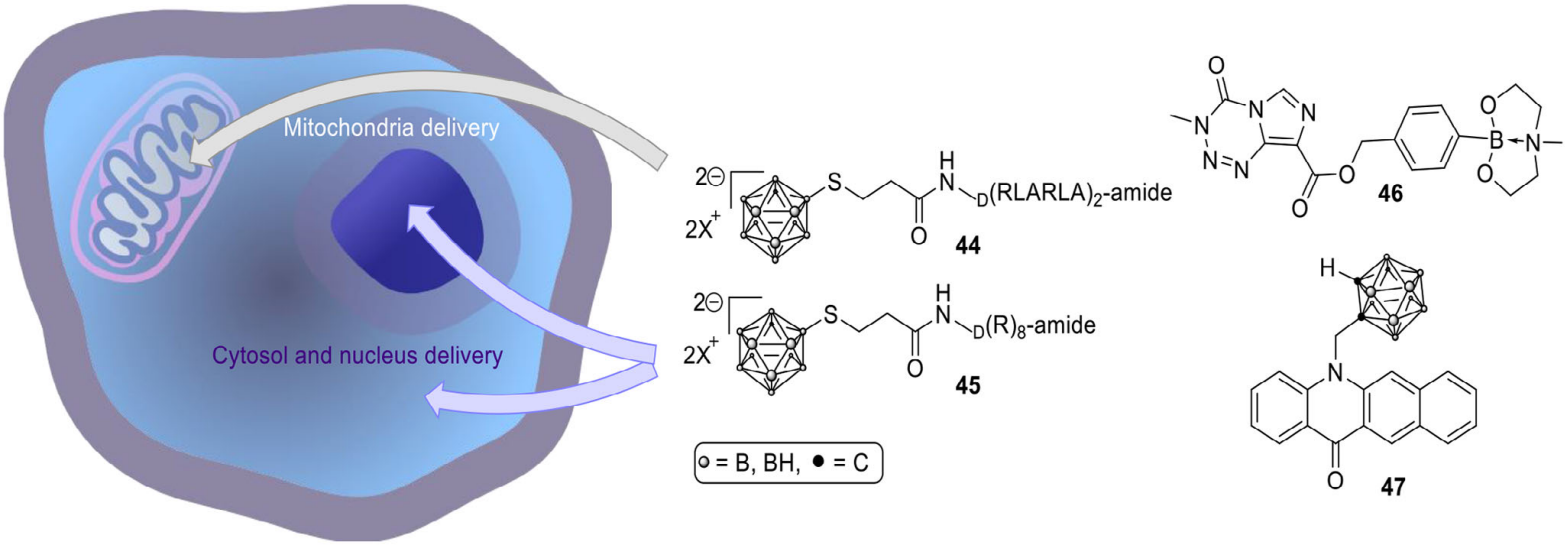
Schematic representation of the intracellular targeted delivery of RLA‐ and R_8_‐peptide conjugated dodecaborates (left) and boron‐containing dual mode chemotherapeutics for BNCT (right) [68 –70].

Targeting subcellular structures such as mitochondria, nuclei, or the cytoskeleton represents a refined approach to enhance the selectivity and potency of BNCT. These strategies aim to deliver boron compounds directly to organelles where radiation‐induced damage can most effectively trigger cell death.

nakase et al. designed cell‐penetrating peptide‐conjugated boron compounds (Scheme 12, **44** and **45**) to achieve controlled intracellular delivery and localization [93]. Their results showed that the organelle‐specific accumulation of dodecaborate significantly improved the efficiency of BNCT by inducing ATP depletion and apoptosis through targeted intracellular mechanisms. This work highlighted that precise subcellular targeting can shape the biological pathways of BNCT‐mediated cytotoxicity.

Building on the concept of combining chemotherapy and BNCT, xiang et al. developed a boronated derivative of temozolomide (TMZB, **46**, first‐line chemotherapeutic that induces DNA lesions by alkylating purine bases) with dual chemotherapeutic and boron delivery properties [94]. TMZB demonstrated similar intrinsic cytotoxicity to temozolomide but achieved higher T/B and T/N (brain tissue) ratios than BPA. In mouse glioblastoma models, BNCT with TMZB achieved markedly greater tumor shrinkage than BPA. The compound retained its DNA‐alkylating capacity, providing cell‐killing effects even in tumor regions with low boron accumulation, thus addressing one of the major limitations of conventional BNCT agents. belchior et al. investigated carboranylmethylbenzo[b]acridone (CMBA, **47**), a boronated acridone that preferentially accumulates in the cytoskeletal fraction of glioblastoma cells [95]. CMBA exhibited strong cellular uptake, low toxicity, and efficient boron incorporation into U87 cells. The authors emphasized that cytoskeletal targeting could provide a complementary route to cell killing, as actin and microtubule disruption are central to tumor progression. Monte Carlo simulations confirmed that over 95% of the absorbed dose originated from boron capture reactions, validating CMBA's potential as an effective and selective BNCT agent.

In summary, mitochondria and DNA‐targeted boron agents expand the therapeutic scope of BNCT by coupling subcellular targeting with chemical or radiological cytotoxicity. Such agents can overcome the limitations of heterogeneous boron distribution and may ultimately enhance BNCT's efficacy against highly resistant tumors such as glioblastoma.

### 3.2 Boronated Polymers, Liposomes, and Other Nanoparticles

Beyond small molecules, a broad range of polymeric and nanoscale carriers is being engineered to concentrate ^10^B in tumors while enabling imaging and safer systemic delivery. These systems often self‐assemble into micelles or vesicles, or form polymer networks, and they can be tailored for receptor targeting, BBB passage, and image guidance.

#### 3.2.1 Boronated Polymers, Micelles, and Vesicles

**SCHEME 13:**
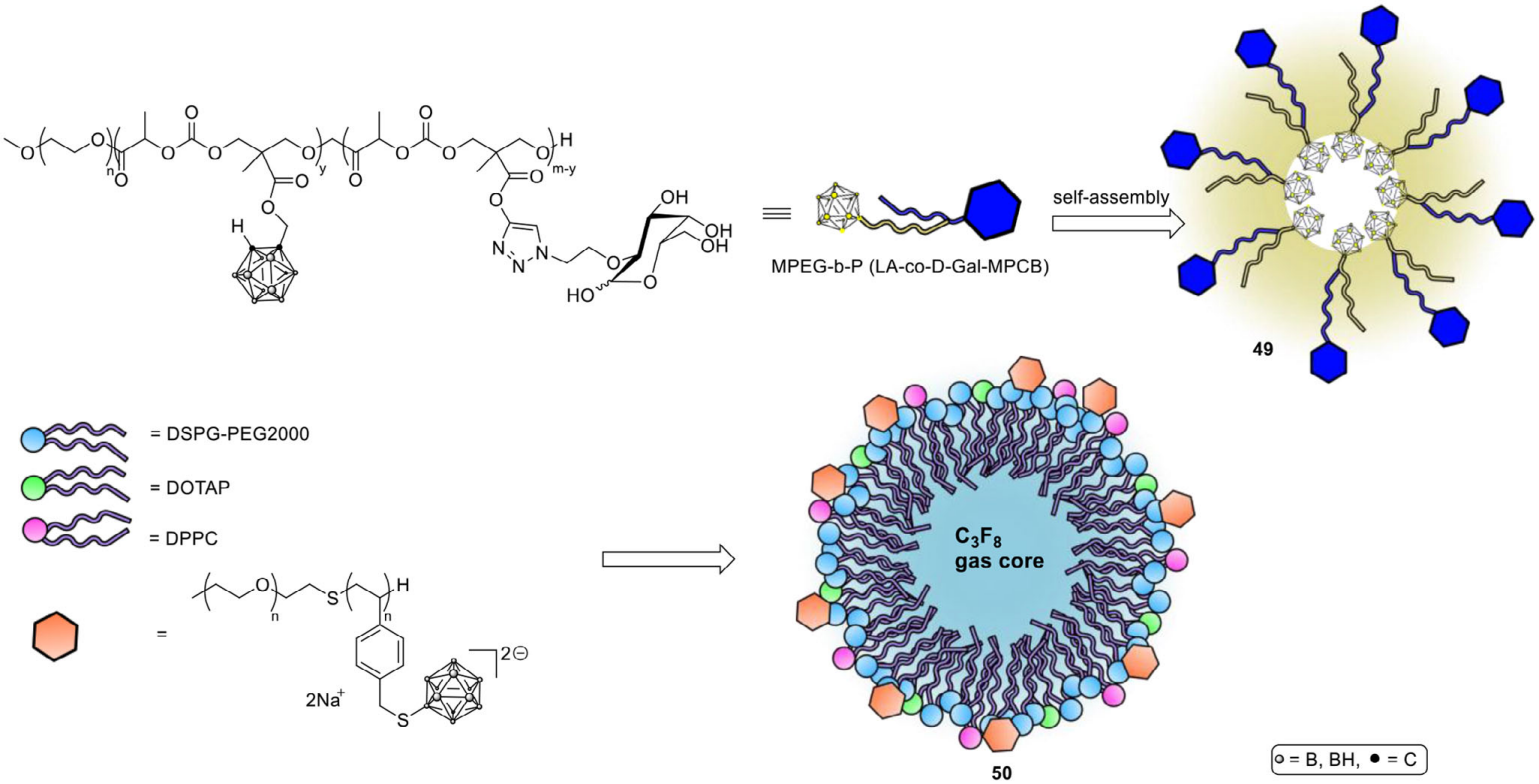
Self‐assembling MPEG‐b‐P (LA‐co‐d‐Gal‐MPCB) micelle (top) and boron‐polymer/microbubble complex for focused ultrasound drug delivery as potential boron carriers for BNCT.

Carbohydrate‐decorated amphiphiles have been used to add active targeting to micelles. PEGylated galactose polymers that load carborane clusters self‐assemble into ∼135 nm micelles (Scheme 13, **49**) and exploit the hepatocyte asialoglycoprotein receptor. In HepG2 (human liver cancer cell line) models they showed low intrinsic toxicity, higher cellular uptake than BSH, and induction of apoptosis upon BNCT. In tumor‐bearing mice, the micelles produced tumor boron levels 4.5‐fold above BSH at 4 h and T/B ratios above 25 at 24 h, with rapid systemic clearance and no evident toxicity [96].

To address delivery across the BBB, polyanionic boron block‐copolymer nanoparticles (**50**) were combined with cationic microbubbles and focused ultrasound. The microbubble‐polymer complexes enabled transient opening of the blood‐tumor barrier and immediate boron delivery to glioma tissue. Compared with a simple mixture of polymer and microbubbles, the pre‐assembled complexes tripled the T/N ratio and more than doubled the T/B ratio minutes after sonication, while maintaining acceptable safety at optimized acoustic pressures [97].

**SCHEME 14:**
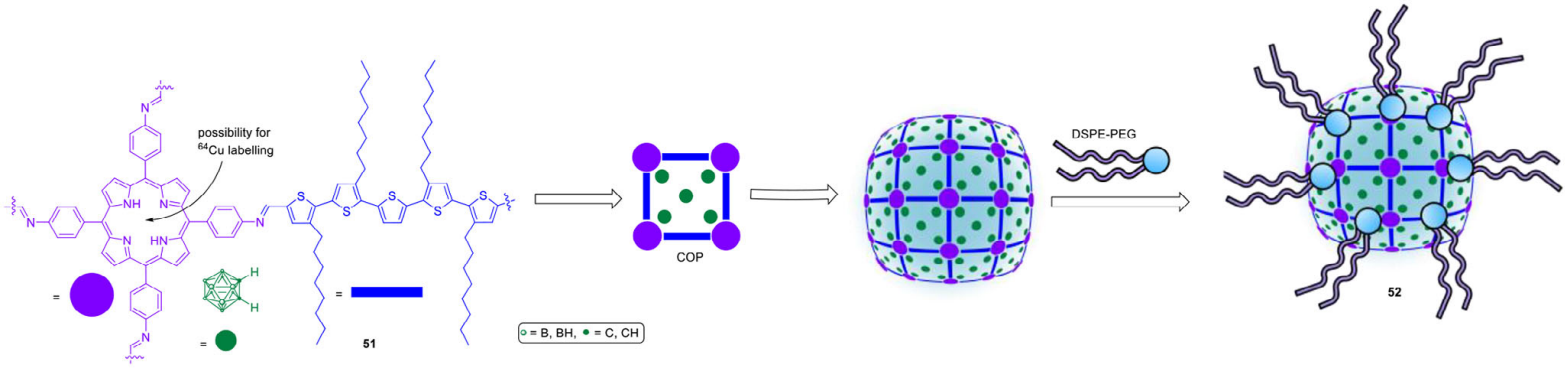
Structure and assembly of a nanoscale imine‐linked covalent organic polymer (COP) carborane carrier for imaging‐guided BNCT [73].

Covalent organic polymers (COP) provide another modular platform. A nanoscale imine‐linked COP was engineered to load carborane (Scheme 14, **51**) and was PEGylated with 1,2‐distearoyl‐sn‐glycero‐3‐phosphoethanolamine‐*N*‐[amino(polyethylene glycol) (DSPE)‐PEG to yield ∼100 nm aqueous nanoparticles (**52**). After ^64^Cu labeling, PET confirmed tumor accumulation, and BNCT with this carrier suppressed 4T1 (breast cancer cell line) tumors without observable tissue damage, illustrating a theranostic route that unites imaging and treatment within a single polymer scaffold [98].

In another study, boron has been embedded directly into a 3D polymer network by crosslinking 2‐hydroxyethylmethacrylate (HEMA) with ^10^B‐enriched boric acid. This poly(HEMA)^10^B material exhibited neutron‐dose‐dependent changes in mechanical properties, modeled by Monte Carlo simulations, and released boron in simulated body fluid. While still preclinical, the work suggests dual roles as a radiosensitive material for dosimetry and as a candidate depot for controlled boron delivery [99].

Building on these strategies, several groups have engineered “smart” nanocarriers that couple high boron payloads with selective entry and retention in tumors. One example uses a cell‐penetrating peptide (transactivating transcriptional activator, TAT) masked by a hyaluronic acid shell. An *ortho*‐carborane‐TAT conjugate self‐assembles into cationic micelles that are then coated with hyaluronic acid to yield core‐shell particles. The hyaluronic acid layer promotes tumor accumulation via CD44 antigen recognition and helps avoid off‐target uptake during circulation. In the tumor microenvironment the shell is shed, exposing TAT to drive cellular entry and increase intracellular boron content to BNCT‐relevant levels in vitro and in vivo [100].

A complementary “polymer‐BPA” concept aims to preserve LAT1 targeting while improving pharmacokinetics. A fructose‐modified PEG‐poly(l‐lysine) forms a reversible boronate complex with BPA. The moderately cationic block copolymer enhances cellular internalization through LAT1‐mediated endocytosis and improves intratumoral retention compared with standard BPA‐fructose, while rapid renal clearance maintains low background in blood and normal organs. In mouse models, this formulation produced higher tumor accumulation contrast and significant growth inhibition after neutron irradiation, and it can be prepared by simple mixing, which is favorable for translation and for pairing with ^18^F‐BPA PET [101].

**SCHEME 15:**
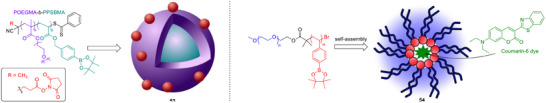
Boronate ester polymer micelles as potential boron carriers for BNCT [77, 80].

Polymerization‐induced self‐assembly has also been used to create sub‐100 nm diblock copolymer nanoparticles with boron‐rich cores (Scheme 15, **53**). These particles, made by reversible addition‐fragmentation transfer (RAFT) polymerization, including versions with phenylboronic acid surface groups, showed encouraging preliminary biocompatibility and establish a scalable route to tune size, morphology, and boron density for future BNCT testing [102].

Beyond soft micelles, boron can be embedded into degradable scaffolds to pair BNCT with local tissue support. Biodegradable polyester films and 3D‐printed matrices were loaded with *closo*‐borates (decaborate or dodecaborate) or filled with a *closo*‐borate hydrogel to yield constructs with controllable mechanical properties, degradation rates, and release profiles. In vitro biocompatibility with osteosarcoma cells and sustained boron release suggest utility for post‐resection bone repair where local BNCT could be advantageous [103].

Furthermore, extracellular vesicles (EV) have emerged as a solvent‐free delivery platform for hydrophobic boron clusters. Using a supramolecular exchange process, carborane (CB) was uniformly loaded into vesicle membranes without damaging vesicle integrity. These CB@EVs penetrated spheroids deeply, accumulated selectively in tumors in vivo, and achieved very high T/N and T/B ratios. BNCT activity in vivo exceeded that of BPA‐fructose and liposomal carborane comparators, while maintaining a favorable safety profile [104].

Recent work has pushed polymeric and supramolecular carriers toward higher boron payloads, tighter tumor selectivity, and better pairing with adjunct therapies. fu et al. reported PEG‐block‐poly(4‐vinylphenyl boronate ester) (PEG‐b‐PVBE) micelles (**54**) of about 40 nm diameter that concentrated boron far more efficiently than BPA in melanoma cells and achieved superior tumor growth delay in vivo, with a higher T/B ratio than the clinical comparator [105]. In a related strategy, chiu et al. prepared boron‐rich PEG‐b‐PVBE micelles that increased cellular boron uptake 13‐fold over BPA and extended tumor growth delay in mice. When BNCT was combined with anti‐PD‐L1 (PD‐L1 = programmed death‐ligand 1) therapy, the effect was further enhanced, with increased T‐cell infiltration supporting an immune contribution to control [106].

Membrane‐derived vesicles offer an alternative to fully synthetic carriers. balboni et al. fabricated nanosized vesicles from patient‐derived glioblastoma cell membranes and loaded them with BSH by sonication or electroporation. The vesicles retained membrane proteins from the parent cells, showed selective uptake into glioblastoma stem‐like cells, and delivered high intracellular boron with negligible uptake by normal oligodendrocyte‐like cells, suggesting a route to tumor‐specific BSH delivery [107].

Supramolecular architectures have also emerged for receptor‐guided delivery. matsumoto et al. developed a fluorophenylboronic‐acid (FPBA) modified polyrotaxane that binds sialic acid on tumor cells. The movable ligand units along the polyrotaxane axle improved multivalent engagement, increased cellular uptake relative to a cellulose control, and raised tumor accumulation and antitumor activity in vivo compared with free FPBA and with standard boron compounds [108]. Finally, dai et al. described lipoic‐acid‐BPA derivatives that reversibly self‐assemble into vesicles under acidic conditions. These vesicles target sialic acid, deplete glutathione, raise reactive oxygen species, and can codeliver doxorubicin with redox‐responsive release. A ^10^B‐enriched version supported BNCT in melanoma and pancreatic cancer models, and sequential BNCT followed by doxorubicin vesicles helped control recurrent disease [109].

Across polymers, membrane‐mimetic vesicles, and supramolecular hosts, newer carriers consistently raise intratumoral boron while improving selectivity and enabling combinations such as immunotherapy or chemotherapy. The next steps are rigorous head‐to‐head comparisons with BPA or BSH in disease‐relevant models and attention to manufacturability, clearance, and safety to translate these gains into clinical studies.

#### 3.2.2 Boronated Biopolymers

Recent work has pushed beyond small‐molecule carriers to biopolymer platforms that can concentrate boron in tumors while adding imaging or targeting functions. A useful conceptual backdrop is the metallacarborane‐DNA field, where modular strategies place boron‐metal clusters onto nucleic acids to create robust, multifunctional biopolymers. That body of work shows how boron clusters can be integrated into biomacromolecules for therapy, sensing, or assembly, and it continues to show new design rules for stability and conjugation in aqueous and biological settings [110].

Within polymer carriers, surface engineering has proven promising. Chitosan‐coated PLGA nanoparticles loaded with *ortho*‐carborane showed higher uptake in B16 melanoma cells than uncoated PLGA, with particles localizing in the cytoplasm near the nucleus and delivering roughly twice as many boron atoms per cell. The chitosan layer increased positive surface charge and enhanced cell association without altering size, pointing to a simple route to boost cellular delivery for BNCT [111]. Building on PLGA, an imaging‐guided approach used PLGA nanoparticles bearing oligohistidine chains to retain a gadolinium‐boron‐theranostic agent for mesothelioma. These particles released cargo in mildly acidic conditions, enriched boron in tumor cells relative to healthy mesothelial cells, and enabled magnetic resonance imaging (MRI) readout paired to neutron irradiation, which reduced clonogenic survival in vitro [112]. In parallel, peptide conjugation has emerged as a general tactic to raise selectivity. A recent review outlines how peptides that target overexpressed receptors can steer boronated constructs to tumors, support combinations with chemo or immunotherapy, and even enable imaging‐guided treatment planning. The authors highlight persistent hurdles in tumor selectivity, intratumoral heterogeneity, and clinical translation, while pointing to targeted peptide‐boron conjugates as a promising way forward [113].

Biopolymer carriers can couple high boron payloads with biological targeting and, in some cases, diagnostic readouts. Progress with PLGA and peptide‐guided systems suggests that tuning surface chemistry and receptor binding will be central to raising tumor selectivity and treatment precision [111, 112, 113].

#### 3.2.3 Boron‐Encapsulated Liposomes

Recent work has focused on engineering liposomal systems that carry high boron payloads while preserving favorable pharmacokinetics and tumor selectivity. One practical advance is the “post‐insertion” approach to PEGylation, which adds PEG after liposome formation. Polyborane‐loaded liposomes prepared this way required only half the PEG‐lipid yet matched conventional pre‐PEGylated formulations in size, surface properties, in vitro behavior, and in vivo tumor uptake, achieving tumor boron levels around 73–78 µg/g and T/B ratios near 5–6 at 24 h [114]. Thermo‐sensitive liposomes have also been explored to release cargo under mild hyperthermia. In glioma models, these carriers improved tumor delivery of BPA and a boronated nitroimidazole, with the hypoxia‐seeking nitroimidazole extending intratumoral boron retention relative to BPA [115].

In a recent study by lee et al., PEGylated liposomes loaded with water‐soluble *nido*‐carborane achieved deep intratumoral distribution, cytoplasmic localization, and robust tumor control in mice after neutron irradiation, with nearly complete growth suppression after one or two BNCT sessions and no notable weight loss [116]. At the delivery level, intra‐arterial administration of transferrin‐targeted PEG liposomes composed of a boron lipid provided about 25 ppm intratumoral boron at day three and produced tumor growth suppression in a rabbit hepatic tumor model following neutron exposure [117]. Parallel efforts are addressing brain delivery: chitosan‐based nanocapsules, including pH‐responsive chitosan‐polypyrrole composites, encapsulated carboranyl delocalized lipophilic cations, showed BBB‐relevant permeability in vitro, high encapsulation efficiency, low toxicity, and selective uptake by glioma cells, supporting their promise as boron vehicles for the application of BNCT for brain tumors [118].

In the work of hirase et al., extracellular vesicles loaded with polyhedral borane and decorated with an oligo‐arginine peptide to trigger macropinocytosis achieved efficient cell entry and strong BNCT cytotoxicity, offering a modular “cassette” EV platform for boron delivery [119]. Building on antibody targeting, carborane‐integrated immunoliposomes prepared through a solvent‐free exchanging reaction were conjugated to anti‐HER2 (human epidermal growth factor receptor 2, HER2) antibodies. In HER2‐overexpressing ovarian cancer models these carriers produced markedly higher cellular boron uptake and a BNCT response roughly fourteen times that of BPA‐fructose, and they suppressed growth in 3D spheroids that mimic tumor extracellular matrix barriers [120].

Liposomal and vesicular systems now span cost‐saving PEGylation, stimulus‐responsive release, receptor and antibody targeting, brain‐directed carriers, and boron‐rich bilayers. Together they point toward clinically adaptable BNCT vectors that improve tumor loading, retention, and therapeutic synergy while maintaining acceptable safety profiles [114, 115, 117, 118, 119, 120, 121]. Beyond encapsulation strategies, increasing attention is now directed toward liposomes in which boron is incorporated directly into the lipid architecture itself, as discussed in the following section.

#### 3.2.4 Boron‐Lipid Liposomes

Formulating boron directly into the lipid phase of vesicles has emerged as a practical route to raise loading and improve stability while preserving nanoscale size and circulation behavior. Early formulation work showed that *ortho*‐carborane can be incorporated into dipalmitoylphosphatidylcholine (DPPC) or distearoylphosphatidylcholine (DSPC) small unilamellar liposomes of roughly 80–100 nm that remain intact in serum, store without major size drift, and retain entrapped markers, establishing a baseline platform for further targeting and in vivo testing [122]. Moving from feasibility to efficacy, an accelerator‐based BNCT study in a U87 glioblastoma model found that PEGylated liposomal BSH produced more durable tumor growth control than BPA or free BSH during the later follow‐up period, supporting the clinical promise of liposomal boron delivery under realistic neutron sources [123]. Related biodistribution work with fluorescently labeled, BSH‐loaded PEG liposomes demonstrated preferential tumor accumulation versus normal brain and provided a rapid microscopy readout to screen liposome uptake across cell lines and in an orthotopic glioma model [124].

**SCHEME 16:**
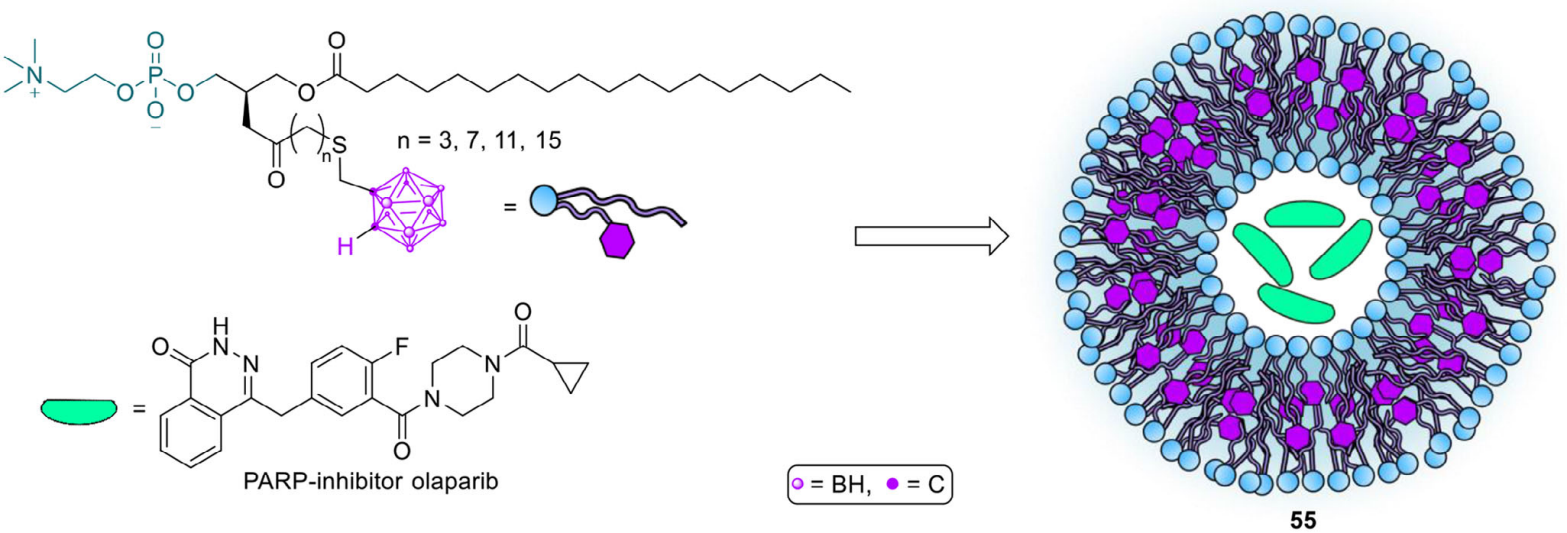
Boron encapsulated chemotherapeutic (e.g., olaparib) loaded in a liposome “boronsome” for combinational chemotherapy and BNCT [121].

Liposome composition itself can be rendered boron‐rich as demonstrated by li et al. with “Boronsome” vesicles (Scheme 16, **55**). In this system, boron is covalently integrated into the lipid bilayer rather than encapsulated in the aqueous core, forming stable biomimetic membranes with high tumor accumulation and prolonged retention as visualized by PET. The boronsomes enabled combined BNCT and chemotherapy, and codelivery of PARP inhibitors further enhanced tumor control by coupling BNCT‐induced DNA damage with impaired repair pathways [121].

**SCHEME 17:**
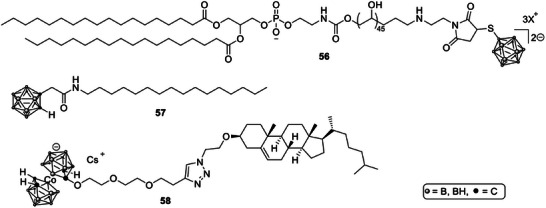
Boron lipids used to construct boron‐rich liposomes for BNCT [100 –102].

Chemistry at the lipid interface is now being used to tune where boron resides and how it is released. A PEG‐based boron lipid that places BSH at PEG termini (Scheme 17, **56**) created “boron‐on‐the‐surface” liposomes which preserved the inner aqueous core for coloading of additional agents and are expected to keep the pharmacokinetics of long‐circulating PEG vesicles while increasing boron payload options [125].

Complementary designs anchor hydrophobic carborane units within the bilayer. Liposomes bearing a simple *closo*‐carborane amide (**57**) inside the membrane reached tumors but with modest selectivity, indicating a need for route optimization or active ligands to raise tumor uptake [126]. A systematic series of cholesterol‐tethered cobalt bis(dicarbollide) (COSAN) lipids (e.g., **58**) showed high loading, excellent encapsulation efficiency, stability after lyophilization and linker‐dependent release without relevant cytotoxicity, suggesting that linker hydrophobicity can be used to balance retention with availability at the tumor site [127].

Finally, synthetic access to boron‐rich lipid building blocks is advancing. New carborane‐containing mono‐, di‐ and triglycerides with up to roughly 19% boron by weight were prepared from readily available precursors. Although acyl migration created isomeric mixtures, the work establishes a versatile route to boron‐enriched lipids intended for future liposomes or micelles for BNCT or proton‐boron concepts [128].

Boron‐lipid liposomes combine high boron loading with the pharmacology of established vesicles. Continued optimization of lipid chemistry, linker design, and active targeting is positioning these carriers for stronger tumor selectivity and more reliable BNCT performance [122, 123, 124, 125, 126, 127, 128].

#### 3.2.5 Boronated Nanotubes, Nanodots, and Nanorods

Boronated nano‐objects such as tubes, dots and rods are increasingly explored as platforms that can concentrate high boron payloads, navigate biological barriers, and present multivalent surfaces for targeting. Many boron clusters and their conjugates behave as nanoscale assemblies in water, which means they should be developed and characterized as nanomedicines rather than only as small molecules. This includes rigorous attention to aggregation state, colloidal stability, and quantitative loading before moving to biological studies. This perspective applies to boronated nanotubes, nanodots and nanorods alike, as each class brings distinct self‐assembly and biological interaction profiles that must be measured and controlled to ensure reliable BNCT performance [129].

##### 3.2.5.1 Boronated Nanotubes

Nanotubular carriers offer high aspect ratios, tunable interiors, and multivalent surfaces that can be engineered for boron loading and intracellular delivery. Two complementary nanotube strategies have recently been explored for BNCT. First, michiue et al. created a simple peptide–boron complex by mixing the cationic (due to lysine side‐chain), self‐assembling A6K peptide (H_2_N‐AAAAAAK‐OH) with the anionic clinical agent BSH [130, 131]. The A6K/BSH formulation formed peptide nanotube‐based complexes that localized to perinuclear endosomal regions in human glioma cells, raised intracellular boron nearly 10‐fold versus BSH alone, and produced tumor‐specific accumulation in a mouse brain tumor model. Upon neutron irradiation, treated cells showed reduced proliferation and colony formation, with no intrinsic cytotoxicity from the carrier. The preparation is operationally straightforward and exploits electrostatic complexation to address poor cellular transduction and nonspecific distribution that limit BSH [131].

Second, heide et al. examined a proteinaceous right‐handed coiled‐coil nanotube (RHCC‐NT) as a modular host for *ortho*‐carborane. Structural analysis and ^11^B{^1^H} NMR confirmed uptake of the hydrophobic cluster into internal cavities where hydrophobic interactions stabilize binding. Molecular dynamics revealed transient interhelical channels that enable entry without disturbing the robust tetrameric architecture, and cell studies showed rapid association and perinuclear accumulation. Because the boron payload is buried, release is not required for neutron activation, and the authors argue that sufficient boron delivery could be achieved by dosing RHCC‐NT‐carborane complexes, with scope for surface functionalization to add tumor targeting [132].

Peptide and protein nanotubes provide orthogonal solutions to BNCT delivery challenges: electrostatic complexation that boosts BSH uptake, and cavity‐hosted loading that solubilizes hydrophobic carboranes while protecting cargo. Continued attention to quantitative loading, microdistribution, and in vivo targeting will determine their clinical potential [131, 132].

##### 3.2.5.2 Boronated Nanodots

Boronated nanodots are typically carbon‐ or boron‐based quantum dots in the size range of 2–10 nm. These structures have gained increasing attention as next‐generation boron carriers for BNCT. Their small size, strong and tunable fluorescence, and high surface functionality make them suitable for both therapy and bioimaging [129].

Early progress in boronated nanodots was achieved by singh et al., who developed boron carbide quantum dots (B_4_C QDs) via a low‐temperature solvothermal route. The resulting ∼7 nm dots displayed bright fluorescence due to structural defect states and exhibited 1.5‐fold higher fluorescence in tumor than in normal cells. The B_4_C QDs were nontoxic, enabled selective bioimaging of cancer cells, and demonstrated promising BNCT performance comparable to clinically used BPA, suggesting their dual potential as both diagnostic and therapeutic agents [133].

feiner et al. introduced a boron‐rich carbon dot system functionalized with tetrazine for click‐chemistry‐based pretargeting in HER2‐positive tumors. These dots exhibited rapid systemic clearance after intravenous injection, minimizing off‐target accumulation, while selective tumor enrichment was achieved through a bioorthogonal reaction with *trans*‐cyclooctene‐modified antibodies. This two‐step pretargeting strategy significantly enhanced tumor selectivity and offers a versatile framework for future BNCT and radionuclide therapies [134].

li et al. designed boron‐containing carbon dots (BCDs) derived from glucose and boronophenylalanine, then encapsulated them into macrophage‐derived exosomes (BCD‐Exos) to improve brain delivery. The hybrid nanodots crossed the BBB and selectively accumulated in orthotopic glioma tissue, reaching 107 ppm ^10^B and achieving T/N ratios exceeding 5, more than double that of BPA. Under neutron irradiation, BNCT with BCD‐Exos resulted in complete survival (100%) of treated mice at 30 days, demonstrating both high efficacy and imaging‐guided delivery through the intrinsic fluorescence of the dots [135].

Further refinement of boron nanodots has emphasized improved biocompatibility and payload. fithroni et al. reported a biodegradable “AB‐type Lactosome” (particles composed of polydepsipeptide linked with polysarcosine (PSar) and PLLA) nanoparticle system incorporating alkylated carboranes. Among the tested derivatives, dihexyl‐*ortho*‐carborane (diC_6_‐Carb) showed the highest loading efficiency and achieved stable tumor accumulation for at least 72 h post‐injection, maintaining favorable T/N and T/B ratios. These data confirmed that diC_6_‐Carb‐loaded lactosomes deliver sufficient ^10^B (> 10^10^ atoms per cell) for therapeutic BNCT doses while avoiding cytotoxicity [136].

Most recently, zhong et al. synthesized human serum albumin‐coated boron‐containing carbon dots (BCDs‐HSA) with 7.1 wt% ^10^B. The HSA shell improved biocompatibility and provided active and passive tumor targeting via albumin‐binding mechanisms. BCDs‐HSA combined high boron content with excitation‐independent orange fluorescence, enabling simultaneous imaging and therapy. In vivo BNCT experiments demonstrated strong tumor suppression in prostate and melanoma mouse models, highlighting the multifunctionality of this system that integrates targeting, imaging, and therapy within a single nanoplatform [137].

Boronated nanodots represent one of the most versatile and multifunctional BNCT carrier types developed to date. Their combination of high ^10^B content, photoluminescence, and tunable surface chemistry enables simultaneous tumor imaging and therapy. Continued optimization of their biocompatibility, targeting, and in vivo retention is expected to advance them toward clinical translation as next‐generation theranostic agents for BNCT. Related strategies in which boron quantum dots are generated in situ from larger, stimulus‐responsive nanoplatforms are discussed separately in section 3.2.6.4. Hybrid and Multifunctional Platforms.

##### 3.2.5.3 Boronated Nanorods

Anisotropic nanostructures such as nanorods offer unique physicochemical advantages for boron delivery, including enhanced cellular uptake and directional energy absorption. An innovative example was presented by pulagam et al., who developed multifunctional gold nanorods (AuNRs) stabilized with polyethylene glycol (PEG) and functionalized with the boron‐rich complex cobalt bis(dicarbollide) (COSAN) for combined boron neutron capture therapy (BNCT) and photothermal therapy (PTT) [138]. The nanorods carried approximately 100 µg boron per mg gold and were radiolabeled with ^64^Cu to enable positron emission tomography (PET) imaging. In vitro studies demonstrated high biocompatibility and dual therapeutic potential, as the nanorods induced cell death upon both neutron irradiation and near‐infrared (NIR) laser exposure. In vivo PET/CT imaging in a gastric adenocarcinoma mouse model confirmed tumor accumulation with T/M ratios around 5.4 at 24 h. Although the boron concentration in tumors (∼0.5 µg B g^−1^) was below the clinical threshold, measurable therapeutic effects were achieved, establishing proof‐of‐concept for synergistic BNCT/PTT using multifunctional boron‐loaded nanorods.

Complementary to this metal‐based platform, li et al. designed dynamic polymeric nanorods via ion coassembly of cationic poly(ethylene oxide)‐block‐poly(glycidyl ethylamine) (PEO‐b‐PGEA) copolymers with *closo*‐dodecaborate clusters [139]. The resulting electroneutral core‐shell nanoparticles exhibited tunable morphologies, i.e. spheres, worms, and rods, with boron contents of 10–20 wt%. Among these, rod‐like particles displayed the highest cellular internalization across glioblastoma (U87) and cervical carcinoma (HeLa) cell lines, highlighting the critical role of particle shape in effective boron delivery.

Together, these studies underline the growing significance of anisotropic nanostructures in BNCT. Both, metallic and polymeric boronated nanorods demonstrate that controlled morphology can improve cellular uptake and enable multifunctional therapeutic strategies, paving the way toward more efficient and versatile BNCT nanoplatforms.

#### 3.2.6 Other Boronated Nanoparticles

The rapid development of boron‐containing nanosystems over the past decade has redefined the scope of BNCT drug design. Research has shifted from optimizing single boron carriers toward creating multifunctional nanoplatforms capable of precise tumor targeting, image‐guided therapy, and controlled release. Recent reviews have highlighted how advances in nanotechnology, particularly in polymeric matrices, inorganic scaffolds, and hybrid composites, enable the integration of boron delivery with complementary therapeutic or diagnostic functions [129, 140].

This new generation of nanocarriers includes biodegradable polymers, mesoporous silica, carbon‐ and nitrogen‐based nanostructures, magnetic and metallic nanoparticles, and hybrid systems that combine these components. Such platforms can achieve boron loadings several orders of magnitude higher than traditional small‐molecule agents while improving pharmacokinetic behavior and minimizing systemic toxicity. Their design often incorporates additional features such as fluorescence or magnetic resonance imaging capabilities, photothermal responsiveness, or stimuli‐sensitive release mechanisms [140].

Collectively, these developments represent a transition from conventional boron drugs toward multifunctional nanomedicines that unify delivery, tracking, and therapeutic activation in a single construct.

##### 3.2.6.1 Polymer‐ and Biopolymer‐Based Nanocarriers

Recent years have seen rapid progress in polymeric boron nanocarriers designed for improved tumor selectivity, biocompatibility, and controlled boron release. soleimanbeigi et al. reported thermo‐responsive chitosan‐PNIPAAm core‐shell nanoparticles conjugated with BPA and methotrexate for glioblastoma therapy. These systems enabled selective boron delivery and temperature‐controlled release near 39°C, with negligible hemolysis and high endocytosis efficiency [141]. Silk fibroin nanoparticles (SFNs) were proposed as natural biopolymer carriers for BSH delivery by bari et al. SFNs significantly enhanced boron internalization in glioma cells, achieving 29.5 ppm boron uptake which is comparable to BPA but at fourfold lower dose [142]. Similarly, poly(vinyl alcohol)/boric acid nanoparticles (PVA/BA NPs) by chan et al. achieved efficient uptake in oral and brain cancer models, delivering up to 70‐fold the required boron concentration for effective BNCT and markedly reducing tumor size in vivo [143, 144].

Boron phosphate nanoparticles enriched in ^10^B and conjugated to anti‐EGFR achieved high intratumoral boron levels and superior in vitro cell kill versus BPA‐fructose in head‐and‐neck cancer models, translating into longer survival and reduced recurrence markers after BNCT in vivo [145].

Targeted polymer nanogels have also emerged. meher et al. designed prostate‐specific membrane antigen (PSMA) targeted PLGA‐PEG nanoparticles loaded with carborane and functionalized for PET imaging in prostate cancer. Although carborane release limited in vivo boron delivery, the study provided a foundation for targeted theranostic BNCT agents [146]. Likewise, BPA‐loaded polydopamine nanoparticles (B‐PDA) by dai et al. achieved efficient BBB penetration, 29.7 ppm boron accumulation in gliomas, and strong in vivo antitumor activity under neutron irradiation [147].

##### 3.2.6.2 Silica‐, Nitrogen‐, and Carbon‐Based Nanostructures

Silica‐based systems continue to dominate nanocarrier design due to their stability, tunable porosity, and surface chemistry. wang et al. synthesized dendritic mesoporous silica nanoparticles conjugated with carborane and decorated with polyethylenimine linked to cyclic Arg‐Gly‐Asp peptide (PEI‐cRGD) for pancreatic tumor targeting. These nanoparticles achieved exceptional boron loading (141.5 mg g^−^^1^) and a T/B ratio of 27.1, far exceeding clinical requirements [148]. vares et al. reported multifunctional fluorescent mesoporous silica nanoparticles (B‐MSNs) cofunctionalized with gadolinium and activatable cell‐penetrating peptides, achieving enhanced delivery to radioresistant chondrosarcoma cells and enabling MRI‐guided BNCT [149]. zhang et al. further advanced this approach using a chitosan‐lactobionic acid‐thioctic acid modified hollow mesoporous silica composite loaded with carborane, showing redox‐responsive release and anti‐inflammatory properties that improved safety and therapeutic selectivity in hepatocellular carcinoma [150]. tang et al. designed a lipid‐bilayer coated MSN decorated with SP94 peptide and loaded with boric acid (37 wt%). The targeted system reached ∼40 ppm tumor boron concentration with T/N and T/B ratios of 4.4 and 5.9, respectively, demonstrating significant therapeutic enhancement compared to BPA [151].

2D nitrogen‐based boron nitride (BN) nanosheets served as dual‐function carriers that deliver ^10^B and release doxorubicin upon neutron exposure, enabling in situ radiochemotherapy with significant tumor growth suppression in a triple‐negative breast cancer model. The platform highlights controlled drug release coupled to BNCT activation and high loading capacity in a single inorganic scaffold [152].

Carbon‐ and nitrogen‐based nanomaterials also showed promise. li et al. introduced phase‐transitioned lysozyme‐coated BN nanoparticles, which protect boron from hydrolysis and allow vitamin C‐triggered degradation after therapy, thereby reducing long‐term toxicity [153]. chiang et al. proposed polymer‐coated boron carbon oxynitride (BCNO) nanoparticles containing ∼30% boron without isotope enrichment, achieving up to 43% in vitro cell death under neutron irradiation and significantly lowering production cost [154]. cudziło et al. synthesized functionalized carbonated boron nitride nanostructures via combustion synthesis, producing highly porous hollow particles with surface hydroxyl and amino groups. These nanostructures displayed good aqueous dispersibility and tunable cell interactions, marking them as versatile candidates for future BNCT systems [155].

##### 3.2.6.3 Magnetic and Other Metal‐Containing Nanoparticles

**SCHEME 18:**
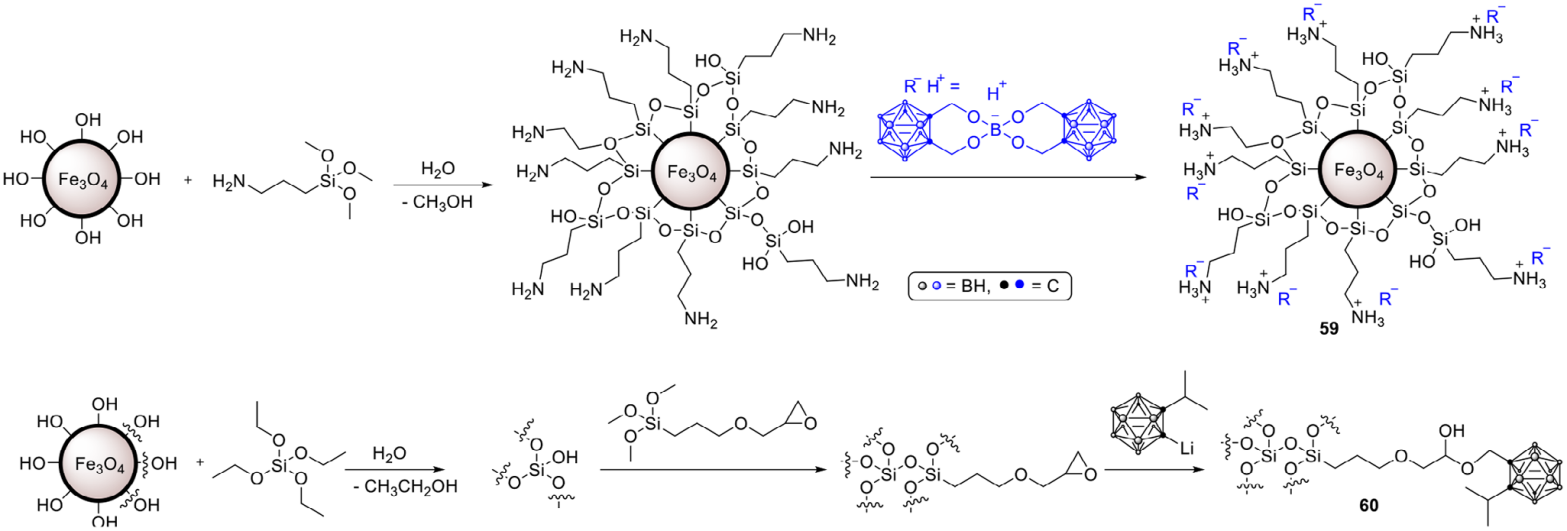
Functionalized Fe_3_O_4_ nanoparticles with organosilane linkers and carborane borates forming biocompatible, superparamagnetic nanocarriers for potential targeted delivery and imaging in BNCT [129].

Magnetic nanocarriers combine targeted delivery with imaging or hyperthermia functions dukenbayev et al. and korolkov et al. functionalized Fe_3_O_4_ nanoparticles with organosilane linkers and carborane borates (Scheme 18, **59** and **60**), yielding biocompatible, superparamagnetic systems stable in physiological media and suitable for magnetic targeting [156, 157]. torresan et al. created iron‐boron nanoparticles (Fe‐B NPs) via laser‐assisted synthesis, which integrated magnetic resonance imaging contrast capability, hyperthermia potential, and boron payload exceeding that of clinical agents by three orders of magnitude. Their gradual lysosomal degradation ensured safe clearance [158].

Magnetically responsive composites were also pursued by makatsaria et al., who produced Fe‐ and Fe_3_O_4_‐doped hexagonal boron nitride nanopowders for magnetically guided BNCT, suggesting Fe_3_O_4_‐doped variants as the optimal compromise between stability and magnetic control [159].

Antibody‐guided gold systems illustrate both the promise and the caveats of metal cores. COSAN‐functionalized, PEGylated AuNPs could be radiolabeled with ^124^I for PET and showed in vivo stability, yet accumulated mainly in liver and spleen with poor tumor uptake, indicating that core geometry and surface engineering remain limiting factors for BNCT delivery [160]. By contrast, HER2‐targeted carborane‐AuNPs were traceable by single photon emission computed tomography (SPECT) and achieved markedly higher T/N (muscle tissue) ratios than nontargeted analogues, underscoring the value of biologic targeting and real‐time pharmacokinetic read‐outs for dose planning [161].

coward et al. reported trimetallic Fe‐B@Au core‐shell nanoparticles, combining boron for neutron capture, iron for magnetic guidance, and a gold shell for peptide attachment. The system achieved high boron incorporation through redox‐transmetalation, offering a flexible platform for multimodal cancer therapy [162]. mikami et al. developed gadolinium borate (GdBO_3_) nanoflakes, introducing a dual boron‐gadolinium approach to combine BNCT with GdNCT and MRI guidance [163].

##### 3.2.6.4 Hybrid and Multifunctional Platforms

A rapidly expanding category involves multifunctional nanocomposites integrating boron with metal‐organic, polymeric, or redox‐active components. wang et al. presented Zr‐TCPP‐based metal‐organic framework (MOF) nanocrystals with exceptionally high boron content (42.5 wt%) that crossed the BBB and delivered 67.5 µg [^10^B] g^−^^1^ to glioma, achieving strong antitumor effects and enabling fluorescence and PET tracking [164]. kawasaki et al. designed phospholipid‐coated boronic oxide nanoparticles that achieved sevenfold higher in vitro BNCT activity than BPA, while a second study introduced hybrid nanogels combining carborane‐bearing pullulan and boron oxide nanoparticles, which provided high tumor selectivity (T/N > 3, T/B > 19) and complete tumor regression without relapse [165, 166].

wang et al. went further by coupling BNCT with chemodynamic therapy and ferroptosis (a Fe‐dependent form of nonapoptotic cell death) using redox‐responsive boron‐iron nanochains (RBNCs) [167, 168]. These hybrid nanostructures disassembled selectively in the tumor microenvironment to release boron quantum dots in situ together with Fe^3+^ ions, thereby enhancing oxidative stress and achieving potent synergistic effects with negligible systemic toxicity. Importantly, the boron quantum dots are not administered as independent nanocarriers but are generated as transient therapeutic intermediates within a multifunctional platform, distinguishing this system from primary boronated nanodot formulations [167].

Finally, elemental and inorganic boron systems also gained traction. Elemental boron nanoparticles stabilized with hydroxyethylcellulose demonstrated stronger cytotoxicity under neutron irradiation than BPA in multiple glioma lines [169]. Such elemental systems may enable low‐cost, high‐density boron delivery once targeted modifications are introduced.

Overall, the diversity and sophistication of nanoparticle‐based boron carriers developed since 2018 underscore this field as one of the fastest‐growing frontier in BNCT drug design. The reported systems, ranging from biopolymer shells and silica frameworks to magnetic hybrids and MOFs, consistently achieve boron concentrations surpassing clinical benchmarks (≥20 ppm) with improved tumor selectivity and functional versatility. Multifunctional nanocarriers integrating imaging, targeting, or combined therapies (chemotherapy, ferroptosis, hyperthermia) now represent a decisive step toward personalized and image‐guided BNCT (see Section 3.4), bridging the gap between preclinical innovation and future clinical translation.

### 3.3 Boron Conjugated Biological Complexes

Here we consider boron conjugated biological complexes as precision carriers that exploit endogenous recognition to enrich ^10^B in malignant tissue while limiting exposure of normal cells. The guiding idea is to couple boron‐rich motifs to peptides, growth‐factor mimics, antibodies, or carrier proteins so that binding, internalization, and retention are dictated by biology rather than passive accumulation. In practice, this means designing conjugates that present sufficient boron payload, maintain target affinity, and display appropriate aqueous behavior and stability during delivery.

#### 3.3.1 Boronated Peptides

Peptide‐boron conjugates have advanced along two complementary lines. One strategy exploits transporter or receptor recognition to enhance cellular uptake and targeting precision, while the other focuses on improving hydrophilicity and serum compatibility so that high boron payloads can be delivered without aggregation or off‐target deposition. Recent progress in this field has established peptides as versatile scaffolds for selective boron delivery, capable of addressing overexpressed receptors, penetrating biological barriers, and even carrying multimodal therapeutic or diagnostic payloads [113].

Transporter targeting has been demonstrated with dipeptides that redirect BPA through the oligopeptide transporter system. BPA‐tyrosine dipeptides enter peptide transporter 1 (PEPT1)‐expressing cancer cells and raise tumor boron after intravenous dosing in a pancreatic xenograft, supporting PEPT1 as a viable alternative when LAT1 expression is limiting [170]. Receptor targeting has focused on prostate and neuropeptide receptors. PSMA‐binding inhibitors outfitted with boronic acids or carboranes retained nanomolar affinity, blocked PSMA in vivo, and delivered 4–7 µg B g^−1^ to prostate tumors with T/M ratios similar to BPA, indicating feasibility but also the need for further optimization to reach therapeutic thresholds [171]. Ligands for G‐protein coupled receptors (GPCRs) have enabled very high carborane loading while preserving function. Human Y1‐receptor selective neuropeptide Y analogs bearing multiple hydrophilic carborane units showed maintained receptor activation and receptor‐mediated internalization with up to ∼80 boron atoms per peptide and low intrinsic cytotoxicity [172]. A gastrin‐releasing peptide receptor agonist was similarly equipped with bis‐deoxygalactosyl carboranes to reduce hydrophobicity, increase metabolic stability, and sustain gastrin‐releasing peptide receptor (GRPR) activation and uptake in PC‐3 cells (human prostate cancer cell line) [173].

Peptide vectors have also been used to enhance intracellular access for otherwise membrane‐impermeant boron clusters. Lipopeptide “pepducin” carriers conjugated to BSH increased cellular uptake in glioblastoma cells and enhanced BNCT radiosensitization compared with BSH alone, with uptake tunable by the lipid and linker composition [174]. Targeting beyond tumor cells to the tumor vasculature is another strategy. A short annexin A1‐binding peptide (IF7) conjugated to either BPA or BSH produced rapid tumor delivery and significant growth control at ultralow doses after multiple BNCT treatments, accompanied by increased CD8^+^ T‐cell infiltration, indicating that vascular targeting also promoted an antitumor immune response [175].

BBB penetration has been addressed with brain‐shuttling peptides. An angiopep‐2 conjugate carrying ^10^B (ANG‐B) increased cellular boron across several lines and outperformed BPA in an intracranial glioma model, achieving higher tumor shrinkage at comparable exposure and maintaining a high T/B‐ratio [176]. Integrin‐targeting cRGD has also been used in nanohybrids to raise tumor retention of BSH in glioma models and to improve T/N ratios after local administration, illustrating how peptide ligands can be layered onto nanocarriers to guide boron delivery to α_v_β_3_‐positive tumors [177].

Methodological work continues to expand the peptide toolbox. A recent synthesis delivered KRGD tetrapeptides (Lys‐Arg‐Gly‐Asp) bearing two carborane units with high boron content and good aqueous solubility while maintaining low cytotoxicity, providing a compact integrin‐addressed motif for future BNCT vectors [178].

Peptide conjugation enables biologically guided delivery of high boron payloads with preserved target affinity and improved pharmacology. Continued work on solubilizing chemistries, brain delivery, and vascular targeting is likely to convert several of these designs into clinically competitive BNCT agents.

#### 3.3.2 Boron Conjugated Antibodies

Antibody‐based delivery systems are being developed to improve the selectivity and cellular uptake of boron agents for BNCT. One recent approach employed a peptide‐boron conjugate that binds to antibodies through the Fc domain (fragment crystallizable region; the tail region of an antibody that interacts with cell surface receptors) to create an adaptable, “cassette‐like” complex for receptor‐targeted delivery. In this system, a Z33 peptide (33 amino acid peptide sequence derived from Protein A) was linked to a dodecaborate cluster and attached to a therapeutic antibody, enabling recognition of tumor‐associated receptors such as EGFR. Upon stimulation with epidermal growth factor, macropinocytosis was induced, which markedly enhanced cellular uptake and improved the biological response after neutron irradiation. This study highlighted that simple membrane accumulation of boron conjugates is insufficient and that active internalization mechanisms are essential for effective BNCT. The modular peptide‐antibody design also allows easy adaptation to different tumor targets, suggesting potential for customizable delivery platforms [179].

Complementary to experimental developments, computational strategies have been introduced to rationally design boronated monoclonal antibodies with preserved structural integrity and target affinity. Using a dedicated in silico approach, selected residues of cetuximab were replaced with boron‐containing analogues to generate a boron delivery antibody capable of maintaining binding to EGFR while introducing therapeutic boron atoms. Molecular docking and dynamics simulations confirmed that the mutations did not disturb the folding of the antibody or the receptor recognition. This approach provides a generalizable framework for engineering antibodies that retain their pharmacological properties while gaining dual action through radiotherapeutic activation during BNCT [180].

#### 3.3.3 Boron Conjugated Proteins

Serum albumin has emerged as a versatile scaffold to increase boron loading, prolong circulation, and incorporate targeting or imaging functionalities. Foundational studies mapped the main conjugation sites of maleimide‐functionalized *closo*‐dodecaborates (MID) on albumin, identifying Cys34 and several lysine residues located in the principal drug‐binding pockets as preferred attachment points, where stable boron‐protein conjugates can form without disrupting the protein's structure or function [181]. Building on this, an α_v_β_3_‐integrin‐addressed construct was created by introducing a cRGD ligand at Cys34 and installing MID on bovine serum albumin (BSA). The resulting cRGD‐MID‐BSA remained noncytotoxic in the absence of irradiation, accumulated more selectively and durably in U87MG tumors than a nontargeted control, and produced significant tumor growth suppression after neutron exposure at a dose of 7.5 mg ^10^B kg^−1^ [182]. Albumin has also been configured as a true theranostic: fluorophore‐ and ^19^F‐MRI‐labeled human serum albumin (HSA) carrying *closo*‐dodecaborate enabled dual‐modality tracking and reduced glioma cell survival after neutron capture while showing little intrinsic toxicity without irradiation, underscoring the feasibility of imaging‐guided BNCT with a single macromolecular carrier [183].

Translational in vivo studies have begun to define dose, timing, and safety windows for albumin conjugates. In a hamster cheek pouch oral cancer model, conjugates of MID and BSA (“MID:BSA”) achieved therapeutically useful tumor boron levels and a significant tumor response with negligible radiotoxicity when administered at 15 mg B kg^−1^ with slow infusion. Higher dosing produced albumin‐related toxicity, highlighting the need to tailor administration to the model and carrier load [184]. In an F98 rat glioma model, a MID‐albumin conjugate (MID‐AC) accumulated and persisted in tumor, afforded survival prolongation comparable to BPA at either 2.5 or 24 h between infusion and irradiation, and achieved a high estimated biological effectiveness, consistent with durable boron availability during beam delivery [185].

**SCHEME 19:**
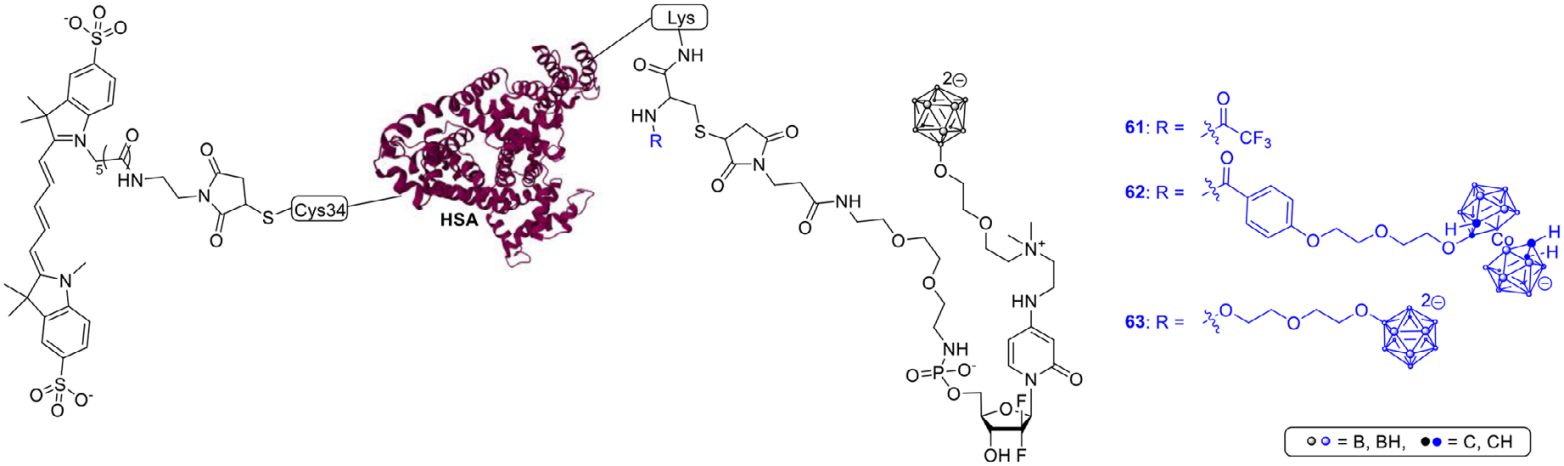
Multifunctionalized serum albumin conjugates with the chemotherapeutic gemcitabine a Cy5 or Cy7 fluorescence handle and a high boron payload. Image based on PDB 1AO6 [156, 157].

Albumin has also been utilized to combine BNCT with chemotherapy. A gemcitabine analogue bearing a *closo*‐dodecaborate was tethered to labeled albumin (Scheme 19, **61,** Image based on PDB 1AO6.) to create a theranostic that retained cytotoxicity against glioblastoma cells and further reduced clonogenic survival under neutron irradiation, indicating a synergistic effect of the chemotherapeutic and boron cluster within one macromolecule [186, 187]. Related constructs that integrate boron‐containing homocysteinamide handles and gemcitabine analogues on albumin (**62**, **63**) show efficient intracellular accumulation. Variants carrying COSAN (**63**) displayed the highest in vitro cytotoxicity and nuclear enrichment, suggesting potential for enhanced efficacy after radiation [188]

Beyond albumin, other protein scaffolds have recently been explored for high‐capacity boron delivery. Apoferritin, a hollow iron‐storage protein with a well‐defined internal cavity, has been exploited as a nanocontainer for metallacarboranes. alberti et al. reported the encapsulation of COSAN within apoferritin via pH‐triggered disassembly and reassembly, yielding a stable Apo:[o‐COSAN]^−^ nanohybrid with high boron content and favorable biocompatibility [189, 190]. The system showed enhanced boron uptake in breast cancer and mesothelioma cells and induced significant neutron‐dependent cytotoxicity, highlighting apoferritin as a versatile protein cage for BNCT applications.

Protein‐mediated delivery strategies have also been investigated using endogenous lipoproteins. Low‐density lipoproteins (LDL), which are actively taken up by many tumors via LDL receptors, have previously been used to transport boron‐ and gadolinium‐containing probes for imaging‐guided neutron capture concepts. While early BNCT‐focused LDL systems predate the temporal scope of this review, more recent work has demonstrated LDL‐based theranostic delivery of anticancer drugs and MRI agents, underscoring the continued relevance of lipoproteins as biocompatible protein carriers that could be adapted for future BNCT translation [191, 192].

Protein conjugation (especially to albumin) supports high boron loading, tumor retention, and the integration of targeting and imaging, and it opens a path to rational combinations with chemotherapy in a single construct. Early in vivo data indicate therapeutic activity with manageable toxicity, warranting systematic optimization of dosing, timing, and payload composition for clinical translation.

#### 3.3.4 Boron Conjugated Viruses and Macrophages

Cellular carriers such as macrophages have emerged as promising vehicles for targeted boron delivery due to their natural biocompatibility, long circulation time, and ability to infiltrate tumor tissue. Their phagocytic activity allows efficient loading of nanoparticulate agents without causing immune rejection, while their tropism for hypoxic and inflamed environments facilitates accumulation in the tumor microenvironment. The use of such immune cells as “Trojan horses” for nanoparticle transport could enhance BNCT by improving tumor selectivity and overcoming physiological barriers. However, effective application depends on maintaining cell viability and migration capacity, and on selecting nanoparticles with optimal size, shape, and surface chemistry to ensure uptake without impairing macrophage function [193].

Recent studies have started to validate this approach experimentally. Boron carbide nanoparticles were shown to interact efficiently with both cell line‐derived and bone marrow‐derived macrophages, with smaller particles inducing less toxicity and better migration performance while maintaining the ability to activate macrophages. These findings indicate that macrophages can serve as suitable carriers for boron carbide nanomaterials, capable of delivering them to tumor sites while minimizing off‐target effects and cytotoxicity [194]. This work supports the concept of macrophage‐mediated BNCT as a form of cell‐based radioimmunotherapy.

In parallel, viral vectors have also been explored as boron delivery systems. The hemagglutinating virus of Japan envelope (HVJ‐E), which possesses strong membrane fusion ability and intrinsic immunostimulatory properties, has been functionalized with boron‐containing polymers to yield hybrid nanocarriers that combine radiotherapeutic and immunotherapeutic effects. Surface modification of HVJ‐E with a biocompatible benzoxaborole copolymer provided stable boron incorporation, preserved viral structure, reduced hemolysis, and supported cellular uptake without toxicity. Such boron‐incorporated HVJ‐E constructs have the potential to couple efficient boron accumulation with immune activation, offering a dual‐action platform for BNCT [195].

Together, these advances illustrate a shift toward biologically active boron carriers that exploit the targeting and immune properties of living systems. Whether through engineered viruses or macrophage‐based “Trojan horse” delivery, these strategies seek to enhance boron localization, enable combination therapy, and move BNCT closer to integrated radiobiological treatment paradigms.

### 3.4 Theranostics and Imaging‐Guided BNCT

Theranostics aims to couple a boron carrier with an imaging handle so that the same construct can reveal where and how much drug reaches the target before irradiation, and in the most advanced concepts even during treatment. Across recent reviews, a shared message is that BNCT will benefit from noninvasive readouts that track boron delivery in space and time and that integrate smoothly with radiation‐oncology workflows [196, 197, 198, 199]. PET already supports patient selection with ^18^F‐FBPA in clinical settings. SPECT and emerging prompt‐gamma SPECT (PG‐SPECT, not part of this review) promise treatment‐time verification linked to the neutron‐capture reaction. MRI can provide high‐resolution anatomy and, when carriers are endowed with paramagnetic or fluorinated tags, a direct signal that cotravels with boron. Fluorescence readouts add cellular and intraoperative visualization that complements tomographic methods [196].

#### 3.4.1 MRI‐Guided BNCT With Paramagnetic or Multimodal Tags

MRI guidance in BNCT is achieved with carriers whose distribution can be monitored by an MRI‐visible signature that indirectly mirrors also the boron localization. Recent reviews highlight two complementary strategies: One couples gadolinium or iron to nanoparticle or macromolecular boron vehicles to generate T1 or susceptibility contrast while retaining therapeutic payloads. The other uses fluorinated motifs to enable ^19^F MRI as a quantitative, background‐free readout that can be co‐registered with anatomical MRI [196, 198].

A representative macromolecular example is the albumin platform, where boronated human serum albumin was equipped with a fluorophore and fluorinated residues for ^19^F MRI. The construct preserved glioma cell viability in the absence of irradiation, allowed multimodal tracking, and produced neutron‐dependent cytotoxicity, illustrating how a long‐circulating protein scaffold can support imaging‐guided BNCT concepts [200].

In addition to nanoscale carriers, MRI‐guided BNCT has also been pursued using low‐molecular‐weight theranostic agents in which gadolinium complexes are directly conjugated to boron‐rich clusters. lanfranco et al. reported a biotin‐targeted carborane‐Gd‐DOTA conjugate (Gd‐AL01) that exploits sodium‐dependent multivitamin transporters for selective tumor uptake [201]. The compound enabled simultaneous BNCT, GdNCT, and MRI visualization, reaching intracellular boron concentrations up to 77 ppm in biotin‐receptor–overexpressing cancer cells and producing clear T_1_‐weighted contrast enhancements. More recently, rakhshan et al. developed a multifunctional sulfamido‐carborane‐Gd‐DOTA construct targeting carbonic anhydrase IX on mesothelioma cells [202]. This low‐molecular‐weight platform combined BNCT and GdNCT with MRI guidance and enzyme inhibition, achieving selective tumor localization, significant growth suppression in vivo, and demonstrating advantages in tissue penetration and clearance compared to nanoparticle‐based systems. These studies highlight that small‐molecule Gd‐B conjugates represent a complementary strategy for imaging‐guided BNCT with favorable pharmacokinetics and precise molecular targeting.

As mentioned in Section 3.2.6.3 (Magnetic and other metal‐containing nanoparticles), iron‐boron nanoparticles produced in a single laser‐assisted step achieved very high boron content and MRI visibility, with additional magnetic features that could assist localization or hyperthermia, and they showed lysosomal degradation compatible with eventual clearance in vivo [158]. Gadolinium‐bearing formulations also demonstrate co‐registration of imaging and therapy. Gd and boron co‐loaded PLGA‐Au nanoparticle assemblies produced T_1_‐weighted signal proportional to gadolinium concentration, and gadolinium and boron levels were positively correlated across preparations. In xenografts, the nanoparticles produced clear tumor hyperintensity and measurable T/M‐ratios, while revealing size‐dependent intratumoral distribution patterns that matter for planning [203, 204].

#### 3.4.2 PET‐Traceable BNCT Theranostics

PET remains the most established imaging technology supporting BNCT, providing whole‐body, quantitative data on tracer uptake that informs patient selection, treatment planning, and dosimetry. The method relies on the radiolabeling of boron carriers or structurally related analogues, allowing clinicians to infer boron biodistribution indirectly through the positron signal. Among these, ^18^F‐FBPA has been the cornerstone tracer for decades. It exploits the same LAT1 system as its therapeutic analogue BPA, thus serving as a reliable surrogate for tumor uptake [205].

Recent reviews emphasize that while ^18^F‐FBPA PET already underpins treatment decisions, further standardization of acquisition and analysis protocols is required to ensure reproducible quantification across centers and scanners [197, 199]. Advanced kinetic modeling and dynamic total‐body PET are expected to refine dose estimation, while low‐cost or hybrid PET/MRI systems may broaden accessibility to image‐guided BNCT [196, 198]. PET does not visualize ^10^B atoms directly but instead reports on carrier pharmacokinetics, which can be integrated with CT or MRI for 3D treatment planning.

Preclinical and translational studies highlight how PET‐based theranostics can actively shape BNCT strategies. A ^64^Cu‐labeled boronated porphyrin nanocomplex (BPN) provided simultaneous fluorescence and PET imaging, revealing that fractionated dosing achieved superior tumor uptake compared to single bolus administration. The optimized regimen yielded intratumoral boron levels exceeding 120 ppm and produced near‐complete tumor suppression after neutron irradiation, demonstrating how PET can dynamically inform therapeutic scheduling [47]. PET‐traceable BNCT beyond amino acid carriers include ^18^F‐labeled sugar‐trifluoroborate conjugates as stable, hydrophilic small molecules suitable for real‐time imaging and potential theranostic use [73]. In prostate cancer, ^89^Zr‐labeled PLGA‐PEG nanoparticles functionalized with prostate‐specific membrane antigen (PSMA) ligands achieved selective tumor accumulation detectable by PET, while also revealing premature boron release in vivo. This is an instructive example of how PET can de‐risk formulations by exposing pharmacokinetic weaknesses early in development [146].

Beyond new tracers, clinical PET research continues to refine quantitative dosimetry. Kinetic modeling with dynamic ^18^F‐FBPA PET in healthy volunteers identified the Ichise MA2 multilinear model as the most accurate method for estimating tissue distribution volume (V_t_), allowing improved calculation of expected boron concentrations in normal organs and informing safer dose constraints for therapeutic BPA‐fructose administration [206]. Translational imaging of thyroid function using [^18^F]NaBF_4_ provided another theranostic paradigm: this simple R‐BF_n_ compound permits concurrent mapping and boron delivery within the same molecular scaffold, suggesting that mixed injections of labeled and unlabeled NaBF_4_ could enable rapid imaging‐planning‐treatment cycles within hours [207].

Proteomic analyses are emerging as complementary tools in this theranostic context. A clinical feasibility study within the EORTC 11001 trial analyzed urine samples before and after BPA or BSH infusion and identified inflammation‐related protein signatures potentially modulated by boron exposure. ferrari et al. proposed urine proteomics as a noninvasive means to monitor molecular effects and treatment response alongside imaging biomarkers, aligning with the growing integration of omics into BNCT personalization [208].

Collectively, PET‐enabled BNCT theranostics illustrate how molecular imaging transforms the therapy from empirical dosing toward precision‐guided intervention. The convergence of pharmacokinetic modeling, multimodal tracer chemistry, and biological biomarker discovery defines a new generation of adaptive, quantitatively controlled BNCT.

#### 3.4.3 SPECT for BNCT

An early example of SPECT‐based BNCT theranostics employed HER2‐targeted, boron‐bearing gold nanoparticles radiolabeled with ^123^I to evaluate tumor uptake by microSPECT/CT. Ex vivo ICP‐MS confirmed boron enrichment in the same lesions, demonstrating how SPECT can validate carrier targeting and retention prior to neutron exposure [161]. This dual readout provides a bridge between tracer‐level pharmacokinetics and therapeutic boron quantification. Recent reviews emphasize that while PET remains dominant for quantitative mapping, SPECT retains practical advantages in versatility and cost. Commonly used isotopes such as ^123^I, ^99m^Tc, and ^111^In can be integrated into boron‐containing molecules or nanoparticle conjugates, providing a flexible route for noninvasive evaluation of new carrier designs [199].

#### 3.4.4 Fluorescence‐Tagged Theranostics in BNCT

Fluorescence imaging provides a straightforward and cost‐efficient complement to nuclear or magnetic techniques for tracking boron carriers in vitro and in vivo. Although its optical penetration limits prevent whole‐body mapping, fluorescence offers real‐time feedback at cellular and subcellular resolution and is particularly valuable in preclinical screening of multifunctional constructs. Recent research demonstrates that boron delivery systems can integrate organic dyes, carborane clusters, and targeting ligands to yield hybrid agents with both diagnostic and therapeutic potential.

One strategy employs polysaccharide‐based assemblies for simultaneous fluorescence tracking and boron delivery. A hyaluronic‐acid nanogel incorporating an aza‐boron‐dipyrromethene (aza‐BODIPY) dye linked to sodium borocaptate achieved strong tumor accumulation and enabled optical monitoring of its biodistribution in vivo [71]. Similarly, a hyaluronate–carborane fluorescent complex exhibited CD44‐mediated tumor uptake and cytotoxicity under neutron irradiation, equaling or surpassing that of BPA‐fructose [70]. These results confirm that fluorescent tagging can facilitate the design of boron‐enriched nanocarriers with built‐in targeting and imaging capabilities.

Albumin conjugates have also been adapted for optical theranostics. A gemcitabine‐*closo*‐dodecaborate analogue tethered to Cy5/Cy7‐labeled human serum albumin combined chemotherapy with BNCT functionality and produced marked cytotoxicity in glioma cells, with a survival fraction of approximately 0.4 after irradiation [186]. Such constructs illustrate how fluorescent monitoring can be integrated into multimodal drug conjugates to visualize pharmacokinetics while enhancing therapeutic efficacy.

At the molecular level, recent efforts have explored carborane modification of fluorophores themselves. zaitsev et al. synthesized a series of carboranyl‐BODIPY conjugates through regioselective substitution Huisgen‐coupling, introducing up to three carborane cages per dye. The *ortho*‐carboranyl derivatives retained fluorescence due to higher affinity for albumin and phospholipid membranes, while *meta*‐carboranyl analogues aggregated intracellularly and exhibited light‐activated lipid peroxidation without necrotic cell death. These findings highlight how the electronic and steric effects of boron clusters can fine‐tune the photophysical and phototoxic behavior of dyes, opening avenues toward dual‐mode agents combining photoactivation and neutron capture [209].

Collectively, fluorescent BNCT theranostics span a spectrum from nanogel‐ and albumin‐based carriers to intrinsically boronated dyes. Their value lies primarily in mechanistic and preclinical domains, enabling direct visualization of cellular uptake, intracellular localization, and retention kinetics of boron agents. As optical techniques are integrated with nuclear and MRI modalities, they will remain a crucial toolset for validating next‐generation BNCT carriers before translation to noninvasive imaging platforms.

## 4 Conclusions, Trends, and Future Perspectives

Despite remarkable chemical innovation, the clinical landscape of BNCT continues to rely exclusively on two boron carrier drugs: BPA and BSH. This dependence reflects a historical interplay between technical accessibility, regulatory familiarity, and limited industrial interest rather than a lack of scientific progress. The preclinical literature over the past decade demonstrates the opposite: a rapidly expanding portfolio of boron carriers with improved tumor selectivity, multifunctionality, and imaging compatibility. Yet none of these next‐generation agents have reached human evaluation. Bridging this translational gap remains the central challenge for the field.

**FIGURE 3:**
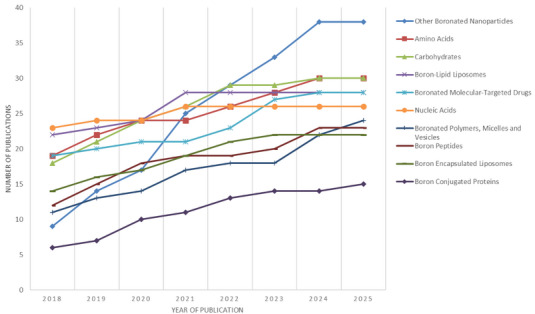
Diagram showing the development of the top ten categories within the scope of this review from 1959 to 2025.

A bibliometric analysis of publications from 2018 to 2025 underscores how research priorities are shifting (Figure 3). The most represented areas are *Other Boronated Nanoparticles*, *Amino Acids*, *Carbohydrates*, *Boron‐Lipids/Liposomes*, *Boronated Molecular‐Targeted Drugs*, *Nucleic Acids*, *Boronated Polymers*, *Micelles and Vesicles*, and *Boron Peptides*. When grouped into broader categories, *Small Molecules* account for 48 publications, *Boronated Polymers*, *Liposomes* and *Other Nanoparticles* for 71, *Boron‐conjugated Biological Complexes* for 25, and *Theranostics and Imaging‐Guided BNCT* for 24. This distribution reflects both the diversification of chemical strategies and the increasing prominence of nanotechnology‐based carriers.

Among these, nanoparticles clearly dominate the current research landscape and likely define the next developmental phase of BNCT. Their modularity allows simultaneous optimization of tumor targeting, pharmacokinetics, and payload capacity [129]. More importantly, nanostructures can be loaded with imaging probes or therapeutic substances, transforming boron delivery systems into true theranostic agents. As emphasized in the recent literature, integrating diagnostic and therapeutic functionality within a single formulation represents a critical step toward individualized BNCT treatment planning [5, 196]. The ability to image boron distribution pre‐ or peri‐irradiation enables real‐time dose adaptation, moving BNCT closer to the standards of precision medicine established in proton and photon radiotherapy.

In parallel, the analysis confirms that small‐molecule research and in there, particularly amino acids, carbohydrates, and molecular‐targeted drugs, remains an essential foundation. The enduring focus on these compounds reflects the clinical legacy of BPA, itself a boronated amino acid, and the practical advantages of simpler pharmacokinetics, scalable synthesis, and established regulatory pathways. Future innovation will likely involve hybrid approaches that merge the proven pharmacology of small molecules with the multifunctionality of nanocarriers, yielding modular platforms compatible with both imaging and therapy.

As highlighted by gozzi et al., many boron‐rich clusters already exhibit colloidal or nano‐scale behavior in aqueous environments, suggesting that the boundary between “molecular” and “nanoparticle” carriers is conceptually fluid [129]. Applying nanomedicine principles such as surface functionalization, size control, and in vivo stability testing to these systems could accelerate their translation by aligning BNCT drug development with the broader pharmaceutical standards of targeted nanotherapeutics [129].

Looking forward, the path to clinical diversification of boron carriers will require coordinated advances beyond chemistry alone. Progress in standardized dosimetry, harmonized clinical protocols, and validated imaging methodologies will be essential to integrate new agents into treatment planning. Equally, public‐sector and industrial investment are needed to offset the high costs of isotopic enrichment, toxicology, and GMP production. In summary, BNCT now stands at a translational crossroads. The chemistry of boron delivery has outpaced its clinical adoption, but the growing convergence of molecular design, nanotechnology, and imaging science provides a clear roadmap toward next‐generation, patient‐personalized BNCT. Achieving this will require the same interdisciplinary collaboration that once enabled BPA and BSH to reach the clinic, but with the scientific maturity and technical infrastructure to bring the next generation of boron carriers across the translational barrier.

## Conflicts of Interest

The authors declare no conflict of interests.
